# A computational framework for the investigation of phosphoinositide regulation

**DOI:** 10.1371/journal.pcbi.1013477

**Published:** 2025-09-30

**Authors:** Hilaire Yam Fung Cheung, Chukiat Tantiwong, Dipali Kale, Jonathan M. Gibbins, Steve P. Watson, Johan W. M. Heemskerk, Albert Sickmann, Robert Ahrends, Joanne L. Dunster

**Affiliations:** 1 Department of Cardiovascular Sciences, College of Medicine and Health, University of Birmingham, Birmingham, United Kingdom; 2 Department of Biochemistry, Cardiovascular Research Institute Maastricht (CARIM), Maastricht University, Maastricht, The Netherlands; 3 Leibniz-Institut für Analytische Wissenschaften-ISAS-e.V, Dortmund, Germany; 4 Institute for Cardiovascular and Metabolic Research, School of Biological Sciences, University of Reading, Reading, United Kingdom; 5 Department of Analytical Chemistry, University of Vienna, Vienna, Austria; Virginia Polytechnic Institute and State University, UNITED STATES OF AMERICA

## Abstract

Phosphoinositides are a group of interconvertible lipids that are located in the membrane of eukaryotic cells. They turnover via complex network of reactions (called the phosphoinositide pathway) that respond rapidly to regulate many aspects of a cell’s response to their environment. Given their low-abundance they are difficult to characterise experimentally. Here we utilise a new experimental method to generate an unusually large dataset that characterises the time-dependent changes in five membrane bound phospoinositides and a soluble inositide in platelet, downstream of its GPVI receptor, where we know the phosphoinositide pathway is particularly active. To shed light on regulatotory steps that are often opaque to experimentation we use this data within a mathematical and computational framework. We construct and assess eleven mathematical models that represent competing interpretations of the dominant mechanisms that regulate the pathway. We find that while four of the models can generate the available data only one model, that incorporates an additional pool of PtdIns, is consistent with the data and is able to successfully predict the effects of an inhibitor. We publish all models openly in a form that is easily usable and adaptable for other researchers to use alongside our or their own data. We studied how changes in the shape and magnitude of events that stimulate the phosphoinositide pathway affect its dynamics. Despite these perturbations, the abundance of Phosphatidylinositol 4,5-bisphosphate (PtdIns(4,5)P_2_) remained stable, consistent with findings reported in the literature.

## Author summary

Cells use signalling molecules called phosphoinositides to control essential functions like movement, shape, and communication. These molecules are present in very small amounts and change rapidly, making them difficult to study. Here, we focused on platelets, blood cells where phosphoinositide signalling is especially active, using data generated by a new experimental method that describes how six different phosphoinositides change over time after stimulation.

To interpret this complex data, we built and tested eleven mathematical models that represent different ideas about how the phosphoinositide pathway is regulated. Most models could reproduce the data, but only one — featuring a secondary pool of a key molecule (PtdIns) — made accurate predictions, including responses to a known inhibitor. This model suggests that internal buffering slows down precursor availability, helping to keep the system stable.

We also found that levels of PtdIns(4,5)P_2_, a critical signalling molecule, remained stable even when stimulation was reduced, highlighting the pathway’s resilience. Our models simplify many aspects of biology but still offer useful insights into how phosphoinositide levels are controlled.

All our models and code are freely available, allowing other researchers to reuse, adapt, and extend them. This work is a step toward better understanding a key signalling system in health and disease.

## 1. Introduction

**Fig 1:**
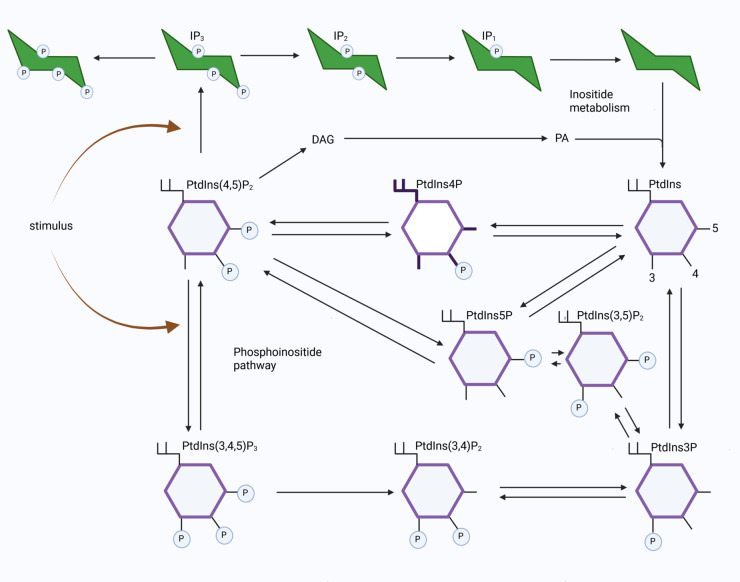
A simplified depiction of the phosphoinositide and inositol pathways. The phosphoinositide species are shown in purple, the inositides in green. Phosphatidylinositol (PtdIns) is shown with the 3’,4’ and 5’ -hydroxy groups indicated, the suffix indicating the total number. Addition and removal of these phosphate groups at these three positions (by kinases and phosphatases) creates seven further phosphoinositide species. The metabolism of inositol phosphate molecules are shown starting with Ins(1,4,5)P_3_ (IP_3_) formation via the hydrolysis of PtdIns(4,5)P_2_ by phospholipase C (the stimulus) that also produces membrane bound diacylglycerol (DAG). IP_3_ is broken down into lower inositol phosphates that are eventually recycled to the membrane via interactions with products generated from DAG, such as phosphatidic acid (PA). Arrows indicate phosphorylation, dephosphorylation or hydrolysis.

Inositol phosphoinositides (PPIs) and their metabolites form a finely tuned network of lipids (depicted in Fig 1) that coordinate many cellular responses in eukaryotic cells, such as cellular signalling, membrane dynamics, and trafficking [1–3]. The membrane bound phosphoinositides comprise a distinct family of eight interconvertible phospholipid subclasses. Their generation is mediated by phosphorylation and dephosphorylation of phosphatidylinositol (PtdIns), which is composed of an inositol head group attached to a glycerol backbone, and two fatty acyl chains that allows it to anchor to cellular membranes. The inositol head group can be phosphorylated at three different positions (numbered 3, 4, and 5), producing the seven additional phosphoinositides: the phosphatidylinositol monophosphates (PtdIns3P, PtdIns4P, and PtdIns5P), bisphosphates (PtdIns(3,4)P_2_, PtdIns(3,5)P_2_, PtdIns(4,5)P_2_), and a trisphosphate (PtdIns(3,4,5)P_3_) [3]. The sequential interconversion and regulation of the eight phosphoinositides is commonly termed the phosphoinositide pathway, the shift in availability of each species in time and space being controlled via a host of lipid kinases and phosphatases that add or remove the phosphate groups. The soluble inositol phosphates are located in a cells cytosol and contain only the insositol ring phosphorylated with one or more phosphates in various combinations. Among these inositides IP_3_ is the most well studied, it is the cytosolic product of receptor-stimulated hydrolysis of the membrane bound phosphoinositide PtdIns(4,5)P_2_ and it mediates Ca^2 + ^ release. It can be phosphorylated to higher or lower phosphorylated inositiols, the lower forms eventually being recycled back to the membrane as PtdIns to participate again in phosphoinositide turnover at the membrane.

Mathematical models have long been used to investigate such dynamical systems as the phosphoinositide and inositol pathways, helping researchers to test hypothesis against experimental data, systematically analyse perturbations and disruptions and help guide further experimental design. Given the ubiquitous role of these lipids in subcellular processes it is unsurprising that they are included, often in reduced subsets, in numerous mathematical models that capture subcellular processes (for example [4–8]). The isolation, identification and quantification of phosphoinositides are challenging because of their low abundance, their amphipathic nature, with two hydrophobic acyl chains and a negatively charged hydrophilic isositol phosphate headgroup [1,9]. Therefore, up until recently, experimental data characterising the changes in phosphoinositide and inositol pathways have been difficult to obtain. This has meant that mathematical models that include PPIs have yet to be compared to large dynamic datasets, instead they have been validated to data that is at steady state, lacking dynamic changes, or is inconsistent, being from many cell types. Of the mathematical models constructed that include many details of the phosphoinositide and inositol pathways, perhaps Olivença et al [10] is the most comprehensive. They focussed on the phosphoinositides and their inter-converting enzymes, adjusting parameter values from literature so that outputs agreed with experimental data describing the steady state values that had been collated from many sources (and cell types). Hille and coworkers [11–13] focused on utilising various models that capture the key steps in G protein–coupled receptors mediated hydrolysis and resynthesis of phosphoinositides to interpret their experimental results generated by Fluorescence Resonance Energy Transfer (FRET), their findings include insight into the robustness of PtdIns(3,5)P_2_ concentrations in response to receptor activation. Suratekar et al [14] constructed models capturing the network underlying synthesis of PtdIns(3,5)P_2_. They compared outputs to their own mass spectrometry data that describes lipid ratios in wild-type and mutant flies, alongside data from other sources. The models supported a scenario where the network supporting PtdIns(3,5)P_2_ synthesis is not closed but includes at least one source and sink. Work by Mazet et al [15] involved the construction of models of key components of the phosphoinositide pathway downstream of platelet thrombin and ADP receptors. While compared to time-dependent data from platelets, the predictions used reactions rates gleaned from multiple literature sources under differing experimental conditions.

Here we wanted to construct a comprehensive model of the phosphoinositide pathway, validated against a single, consistent and comprehensive dataset, that could be used as a formal representation of the current understanding of how the phosphoinositide pathway functions. This is in the expectation that the model can be used and adapted by researchers, modifying to their own experimental setups and cell types as knowledge and data evolve. We focus on the pathway as it occurs within platelets, downstream of one of its principal receptors, glycoprotein VI (GPVI).

Platelets are small anuclear blood cells that are of great importance in haemostasis, their activation and subsequent aggregation into platelet plugs underlie blood clots that stop bleeding. But, blood clots formed inappropriately also play a critical role in coronary artery diseases and platelets contribute to many other physiological processes including inflammation, wound healing and antimicrobial host defence. A platelet’s lack of a nucleus (removing the complexities of gene transcription and translation) make them an ideal focus for mathematical models of subcellular interactions [16]. The phosphoinositide pathway is known to be highly active in platelets [17,18]. Its central role in platelet activation being consistent with mutations in the enzymes responsible for their synthesis and degradation having been linked to a variety of diseases and phosphoinositide 3-kinase inhibitors having been proposed as novel antiplatelet agents to prevent thrombotic events in stroke and cardiovascular diseases [19]. The evidence of the importance of the phosphoinositides to a platelet’s response has spurred the development of methods capable of measuring changes in these low-abundant lipids [9]. Here, we take advantage of this by developing models of the phosphoinositide pathway and inferring and validating the models against experimental data collected under this new workflow. The dataset is unusual not only in its consistency, describing the changes in lipids within this single cell type under consistent experimental conditions, but also in its density, capturing the dynamic changes (measured at seven individual time-points) in five phosphoinositides and an inositide. With the aim of increasing understanding of the mechanisms that dominate the regulation of the phosphoinositide pathway in platelets eleven alternative models were constructed, each including differing methods of regulation. The models were systematically inferred from, and compared to, the experimental data using a computational framework that allows for the comprehensive testing of beliefs in the evidence of data. It provides, over other techniques such as genetic algorithms and simulated annealing, the estimation of kinetic parameters (rather than reliance on single point values from literature) and information on uncertainties both in the parameter values and in model predictions. Models able to replicate the experimental dataset were checked for their ability to constrain rates of reactions, make biologically feasible predictions for experimentally opaque components before being validated against additional data describing the effect of an phosphatidylinositol 4-kinase inhibitor with further investigations predicting the effect of varying the shape and magnitude of stimulating events.

The structure of this work is as follows. In Sect 2 we describe the methods used to collect the data, the development of the eleven mathematical models and the computational framework used to integrate the models to the experimental data. In Sect 3 we discuss the results, reporting on the data, which models are able to generate it and why. We then validate the models against a new dataset generated under the effect of an inhibitor and predict the effects of changes in the signalling events that trigger changes in the pathway. We conclude in Sect 4 with a discussion. To facilitate use of the models by researchers who may be unfamiliar with mathematical techniques publicly available code to run the models in the format of R notebooks is available.

## 2. Methods and model

### 2.1. Experimental data

The experimental data describes the changes within human platelets downstream of the principal collagen receptor GPVI. The data is time-dependent, characterising the levels of one soluble inositide (IP_1_) and five of the eight phosphoinositides (PtdIns, PtdIns4P, PtdIns(4,5)P_2_, PtdIns(3,4,5)P_3_ and PtdIns(3,4)P_2_), these being the most abundant and easiest to detect. This initial dataset is supplemented with data characterising the surrounding signalling events that stimulate changes in the phosphoinositides. A second, smaller dataset is then generated, under the effect of an inhibitor, and used for model validation.

The phosphoinositides were measured via a newly developed method that utilises a quantitative targeted ion chromatography-mass spectrometry-based workflow, described in [9], that separates phosphoinositide isomers and increases the quantitative accuracy of measured phosphoinositides. In summary washed platelets from healthy donors were pretreated with apyrase and indomethacin, and subsequently stimulated with CRP. High concentrations of collagen related peptide (CRP, Cambcol Laboratories), at 30μg/mL, was used to improve the detection of the low abundance phosphoinositides, especially PtdIns(3,4,5)P_3_. Stimulation was stopped at specified time points (0,30,60,90,120,180 and 600s), with ice cold 1M HCI [20] and the samples were analysed and quantified using ion chromatography tandem mass spectrometry system.

To characterise changes in the inositol 1-phosphate washed platelets (at 8 × 10^8^ cells/mL) were pre-treated for 10 min with vehicle and measured using IP-ONE ELISA (Cisbo) in the presence of apyrase (2.5 U/mL), indomethacin (20 *μ*M) and 50 mM LiCl. The concentration of platelets, inhibitors and LiCl was based on similar studies and manufacturer’s instructions. [21,22].

Stimulation of a platelet through the GPVI receptor initiates many downstream signalling events that predominantly stimulate the phosphoinositide network through Phospholipase C (PLC*γ*2), which acts preferentially on the polar head group of the phosphoinositide PtdIns(4,5)P_2_ to generate the soluble inositol IP_3_, and phosphatidylinositol 3-kinase (PI3K) which catalyses PtdIns(4,5)P_2_ to PtdIns(3,4,5)P_3_. To characterise these stimulating events time-courses of phosphorylation of Spleen tyrosine kinase (Syk), the adaptor protein linker for activation of T cells (LAT), at Y200, Bruton’s tyrosine kinase (Btk) and PLC*γ*2 (Y1217) were measured, the phosphorylation of LAT being chosen as a proxy for PI3K which it recruits and activates [23]. In summary, washed platelets (at 4 × 10^8^ cells) were stimulated with 30μg/mL CRP in the presence of 9μM eptifibatide, and were lysed at the stated time after the addition of CRP. The cell lysates were probed against phospho-specific antibodies to determine the extent of tyrosine phosphorylation. The addition of eptifibatide prevented the interference of integrin *α*IIbβ3 outside-in signalling, which acts through Syk, and hence prevented platelet aggregation under the stirring conditions [24].

Similar experiments to these conducted above where carried out to determine the effect of 1 *μ*M GSK-A1 on CRP-induced accumulation of IP_1_ and the phosphoinositides PtdIns4P and PtdIns(4,5)P_2_.

### 2.2. Model development

**Fig 2:**
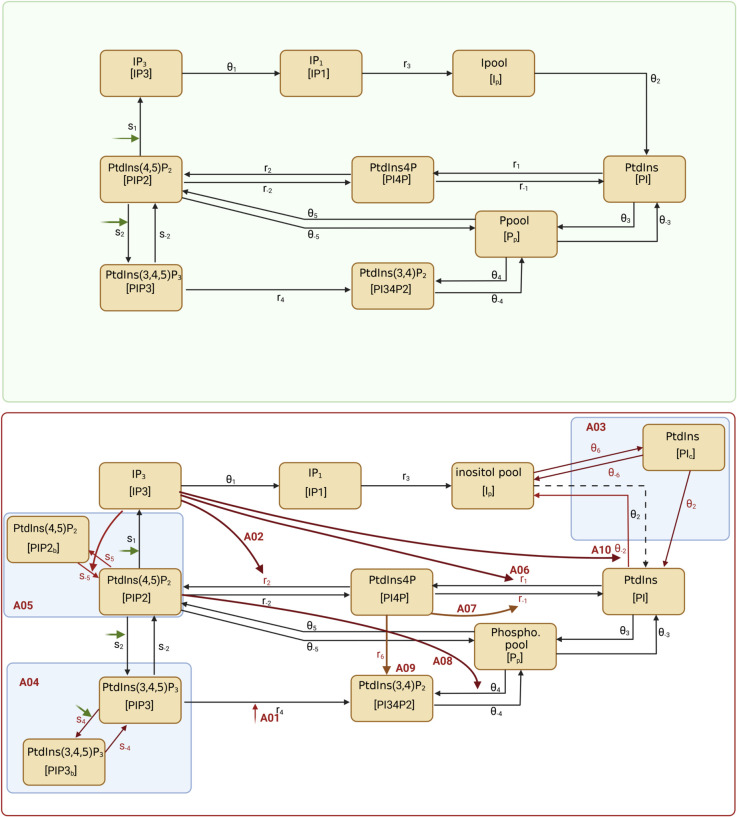
A schematic of the reactions captured in the original model (named A0) of the phosphoinositide pathway (top) and modifications (bottom) which are summarised in Table 3. Boxes indicate the phosphoinositides and inositols included in the models, the corresponding model variables being shown in brackets. Arrows connecting boxes represent conversion from one species to another, arrows directed at lines represent processes that promote a reaction with those in green depicting promotion of the pathway by surrounding signalling events captured in *s*(*t*). Model names and arrows shown in red are reactions introduced in the modification (marked alongside). The broken line represents a reaction that is removed in model A03. Dimensional parameters associated with particular processes are placed next to the relevant arrows. These are described in Table 2 and the model variables in Table 1. Created with BioRender.com.

The first step in the modelling process is to organise the key components and known interactions into a network diagram. We start by refining the steps presented in Fig 1, excluding parallel pathways (such as the resynthesis of DAG) and intermediate steps for which we have no experimental data. We aim to simulate the time-dependent changes of the PPIs and their metabolites within a single platelet, matching the spatially averaged experimental data. We ignore regulation of the pathway via spatial effects and the phosphoinositide kinases and phosphatases that add and remove phosphate groups, investigating their effects with later model modifications. There are eight phosphoinositides, we have experimental data describing the dynamics for five of these, and include these in the model. The remaining three interconverted low abundance species (PtdIns5P, PtdIns(3,5)P_2_ and PtdIns3P) for which we have no data are captured by a single variable (*P*_*p*_). In platelets, signal transduction through the GPVI receptor leads the activation of the protein Syk, the formation of a signalosome centred around the transmembrane adaptor protein LAT, and the recruitment of PI3K and Btk [25]. The latter proteins play a role in facilitating PLC$\gamma_{2}$ activation that leads to the cleavage of PtdIns(4,5)P_2_ in to the second messengers diacylglycerol (1,2-DAG) and the Inositol 1,4,5-triphosphate (IP_3_), PI3k also phosphorylates PtdIns(4,5)P_2_, generating PtdIns(3,4,5)P_3_ [23]. Because of its central role in signal transduction, PtdIns(3,4,5)P_3_ is generated exclusively by PI3K-mediated phosphorylation of PtdIns(4,5)P_2_ and cannot be formed from PtdIns(3,5)P_2_ or PtdIns(3,4)P_2_. We neglect these upstream signalling events and instead model stimulus through these proteins with a function (*s*(*t*)) that captures the qualitative form of data showing phosphorylation of Syk, LAT, Btk and PLC$\gamma_{2}$ (see Fig A in S1 Text). The generation of IP_3_ has a direct influence on Ca^2 + ^ flux and its inositol derivatives (including IP_1_), which are recycled, forming PI before being re-incorporated into the plasma membrane. In our model we include a valid but simple form of inositol metabolism, capturing IP_3_, neglecting its slow metabolism to higher forms but including its faster conversion to IP_1_ (for which we have data), which is a subsidiary step (with variable *I*_*p*_) that precedes the return to PI. This simplistic view of the phosphoinositide pathway, that we hereafter call A0, is depicted in Fig 2 (top panel), a summary of the model variables is given in Table 1 and the parameters in Table 2. The model, described by the following equations, uses ordinary differential equations of second order and relies on mass action kinetics due to its mechanistic simplicity and generality, avoiding Michaelis-Menten, power-law, or S-system formulations that require additional assumptions or parameters not accessible by available data, a useful review of alternative approaches is given by Voit [26].

$$\frac{d[PI]}{dt}=\theta_{2}\;[I_{p}]-r_{1}\;[PI]+r_{-1}[PI4P]-\theta_{3}\;[PI]+\theta_{-3}\;[P_{p}],$$ (1a)

$$\frac{d[PI4P]}{dt}=r_{1}\;[PI]-r_{-1}[PI4P]-r_{2}\;[PI4P]+r_{-2}\;[PIP2],$$ (1b)

$$\frac{d[PIP2]}{dt}=r_{2}\;[PI4P]-r_{-2}\;[PIP2]-s_{2}s(t)\;[PIP2]+s_{-2}\;[PIP3]-s_{1}s(t)\;[PIP2]+\theta_{5}[P_{p}]-\theta_{-5}\;[PIP2],$$ (1c)

$$\frac{d[PIP3]}{dt}=s_{2}s(t)\;[PIP2]-s_{-2}\;[PIP3]-r_{4}\;[PIP3],$$ (1d)

$$\frac{d[PI34P2]}{dt}=r_{4}\;[PIP3]+\theta_{4}[P_{p}]-\theta_{-4}\;[PI34P2],$$ (1e)

$$\frac{d[P_{p}]}{dt}=\theta_{3}\;[PI]-\theta_{-3}\;[P_{p}]-\theta_{5}[P_{p}]+\theta_{-5}\;[PIP2]-\theta_{4}[P_{p}]+\theta_{-4}\;[PI34P2],$$ (1f)

$$\frac{d[IP3]}{dt}=s_{1}\;s(t)\;[PIP2]-\theta_{1}\;[IP3],$$ (1g)

$$\frac{d[IP1]}{dt}=\theta_{1}\;[IP3]-r_{3}[IP1],$$ (1h)

$$\frac{d[I_{p}]}{dt}=r_{3}[IP1]-\theta_{2}\;[I_{p}].$$ (1i)

**Table 1:** Variable names used in the mathematical models. With descriptions of their biological counterpart, initial conditions (copy numbers) and their source. All units are molecules platelet^−1^. Additional variables [*PI*_*c*_], [*PIP*2_*b*_] and [*PIP*3_*b*_] are included in models A03, A05 and A04 respectively. In model A03 the initial condition for PtdIns is assumed to be split evenly between *PI* and newly introduced [*PI*_*c*_]. In model A05 the initial condition for PtdIns(4,5)P_2_ is assumed to be split evenly between [*PIP*2] and [*PIP*2_*b*_]. In model A04 the initial condition for [*PIP*3_*b*_] is set to zero.

Variable	Description	Initial conditions	Source
[*PI*]	PtdIns	2,700,000	In house and [27]
[*PI*4*P*]	PtdIns4P	64,000	In house
[*PIP*2]	PtdIns(4,5)P_2_	310,000	In house
[*PIP*3]	PtdIns(3,4,5)P_3_	1,900	In house
[*PI*34*P*2]	PtdIns(3,4)P_2_	5,200	In house
[*P*_*p*_]	Pool of unmeasured phosphoinositides	25,000	[28],[29]
[*IP*3]	Inositol trisphosphate	0	In house
[*I*_*P*_]	pool of unmeasured Inositol	100,000,000	[30]
[*IP*1]	Inositol phosphate	0	In house

**Table 2:** Parameter descriptions. All models use the first seventeen parameters, except model A03 where parameter *θ*_2_ represents the reaction $\left[PI_{c}]\to [PI\right]$, the final eight parameters placed after the break ($\theta_{6}$ through to $\theta_{-2}$) are introduced in the model indicated in brackets alongside the relevant reactions. All units are sec^−1^ except where indicated by asterisks. *r*_1_, *r*_−1_, *r*_2_ and $\theta_{4}$ has units sec^−1^ in all models except for A06, A07, A02 and A08 respectively where units are molecules platelet^−1^ sec^−1^. Parameter *s*_−5_ is introduced in model A5 and parameter $\theta_{-2}$ in model A10 where their units are molecules platelet^−1^ sec^−1^. A graphical depiction of the interactions that these parameters govern is given in Fig 2.

parameter	reaction	parameter	reaction
*r*_1_ (*)	[PI]→[PI4P]	$\theta_{-5}$	[PIP2]→Pp
*r*_−1_ (*)	[PI4P]→[PI]	*s*_1_	[PIP2]→[IP3]
*r*_2_ (*)	[PI4P]→[PIP2]	*s*_2_	[PIP2]→[PIP3]
*r*_−2_	[PIP2]→[PI4P]	*s*_−2_	[PIP3]→[PIP2]
*r*_3_	$\left[IP1]\to [I_{p}\right]$		
*r*_4_	[PIP3]→[PI34P2]	$\theta_{6}$	[Ip]→[PIc] (A03)
*θ*_1_	[IP3]→[IP1]	$\theta_{-6}$	[PIc]→[Ip] (A03)
*θ*_2_	[IP1]→[PI]	*s*_4_	$\left[PIP3]\to [PIP3_{b}\right]$ (A4)
$\theta_{3}$	$\left[PI]\to [P_{p}\right]$	*s*_−4_	$\left[PIP3_{b}]\to [PIP3\right]$ (A4)
$\theta_{-3}$	$\left[P_{p}]\to [PI\right]$	*s*_5_	$\left[PIP2]\to [PIP2_{b}\right]$ (A5)
$\theta_{4}$ (*)	$\left[P_{p}]\to [PI34P2\right]$	*s*_−5_ (*)	$\left[PIP2_{b}]\to [PIP2\right]$ (A5)
$\theta_{-4}$	$\left[PI34P2]\to [P_{p}\right]$	*r*_6_	[PI4P]→[PI34P2] (A9)
$\theta_{5}$	$\left[P_{p}]\to [PIP2\right]$	$\theta_{-2}$ (*)	$\left[PI]\to [I_{p}\right]$ (A10)

This is a conserved system where

$$s(t)=(a_{1}t\exp(-a_{2}t^{2})+a_{3}\tanh(a_{4}t))$$ (2)

captures the time-dependent dynamics of upstream events. The parameters of *s*(*t*) are adjusted to capture the qualitative shape of phosphorylation following stimulation with CRP (see Fig A in S1 Text for details). The parameters of this function can be adjusted so that model simulations can be generated under different forms of stimulation of the pathway, such as the changes to the magnitude and shape that are investigated in Sect 3.5.

Based on literature we produce a list of alternate biological hypotheses that capture different modes of regulation of the phosphoinositide pathway, some of these describe regulation of the pathway via the action of phosphoinsitide kinases or phosphatases while others attempt to introduce means of sequestration or spatial effects. These hypotheses are labelled A01-10, they are depicted graphically in Fig 2 (bottom panel) and are described in detail in Table 3. The full equations for the alternative models are provided in S1 Text (Sect S1).

**Table 3:** Summary of modifications to the model A0. These are depicted in Fig 2, bottom panel. Full equations detailing all modifications are given in S1 Text. UOM - unit of measure (see Table 2 for their values); PM - plasma membrane; PH - pleckstrin homology.

Model	Description
A01	**The role of SHIP1/2 in promoting the conversion of PtdIns(3,4,5)P_3_ ([PIP3]) to PtdIns(3,4)P_2_ ([PI34P2]).** Activation of platelets with thrombin has been shown to lead to the translocation of SHIP1/2 to the actin cytoskeleton where they promote the dephosphorylation of PtdIns(3,4,5)P_3_ to PtdIns(3,4)P_2_, this being supported by evidence from SHIP-1 knockouts where thrombin activation leads to the accumulation of PtdIns(3,4,5)P_3_, and reduced production of PtdIns(3,4)P_2_ [31]. SHIP-1 phosphorylation has been shown to occur rapidly in platelets after stimulation with CRP [32]. Implementation introduces no new parameters. Stimulation (through the function *s*(*t*)) now promotes *r*_4_. Eqs (1d,1e)
A02	**The role of PKC and Rac1 in regulation of the synthesis of PtdIns4P.** Activation of protein kinase C by Ca^2 + ^ has previously been shown to lead to the phosphorylation and activation of Rac1, a small GTPase, which in turn translocates the kinase PIP5K to the actin cytoskeleton where it can promote phosphorylation of PtdIns4P to PtdIns(4,5)P_2_ [33]. No new parameters are required to implement this modification. Variable [*IP*3] is used as a surrogate for Ca^2 + ^ and Rac1 activation, where it now promotes *r*_2_ (UOM adjusted). Eqs (1b,1c)
A03	**The spatial distribution PtdIns (PI).** PI, the precursor of phosphoinositides, is thought to be synthesized primarily in the inner membrane of the endoplasmic reticulum (the dense tubular system (DTS) in platelets) [34,35]. This precursor of the other phosphoinositides is then thought to be delivered to other membranes either by vesicular transport or via cytosolic PI transfer proteins, with PI transfer proteins (PITP) having been shown to facilitate the transfer of PI from one membrane to another in platelets [36]. Implementation introduces 1 new variable [*PI*_*c*_], that represents PI in the DTS, and 2 new parameters ($\theta_{6}$, $\theta_{-6}$). Eqs (1a,1i)
A04	**The stabilisation of PtdIns(3,4,5)P_3_ through PH binding domains.** PtdIns(3,4,5)P_3_ regulates an array of PH domain-containing proteins, including bruton tyrosine kinase (Btk) which plays a role in platelet activation through GPVI [37–39]. We hypothesize that the binding of Btk to PtdIns(3,4,5)P_3_ may disrupt PtdIns(3,4,5)P_3_ synthesis [39]. Implementation introduces 1 new variable [*PIP*3*b*] representing PtdIns(3,4,5)P_3_ bound to Btk and 2 new parameters (*s*_4_, *s*_−4_), the stimulus promotes *s*_4_. Eq (1d)
A05	**The binding and sequestration of PtdIns(4,5)P_2_.** Myristoylated alanine-rich C kinase substrate (MARCKS) is known to attach to the plasma membrane of quiescent cells where it protects and sequesters PtdIns(4,5)P_2_ until cytosolic calcium increases lead to MARCKS shape change and the release of PtdIns(4,5)P_2_ [40,41]. Implementation introduces 1 new variable [*PIP*2_*b*_] representing MARCKS bound [*PIP*2] and 2 new parameters (*s*_5_,*s*_−5_), using variable [*IP*3] as a surrogate for Ca^2 + ^ which promotes *s*_−5_. Eq (1c)
A06	**PtdIns4P kinase regulation.** Membrane contact sites (MSCs) are formed by Ca2+ recruitment of proteins such as E-Syts, ORPs, bringing the plasma membrane and inner membrane close together [35]. Once formed PIP lipid transfer proteins and PIP kinase/phosphatase are in close proximity, which we hypothesise fuels the resynthesis of PtdIns4P from PtdIns to enable replenishment of PtdIns(4,5)P_2_. Implementation uses [*IP*3] as a surrogate for Ca^2 + ^ and the recruitment of proteins that promote *r*_1_ (UOM adjusted). No new parameters.
A07	**The ability of Osh proteins to promote dephosphorylate of PtdIns4P.** The oxysterol-binding homology (Osh) proteins, which localize to the PM dependent upon PtdIns4P levels, has been shown to regulate the activity of Sac1, a phosphatase that can dephosphorylate PtdIns4P back to PI [42]. Implementation allows [*PI*4*P*] to promote *r*_−1_ (UOM change).
A08	**The role of Ptdlns 4-kinase.** An enzyme which phosphorylates PtdIns3P to PtdIns(3,4)P2 has been found in platelets [43]. Subsequently PtdIns(4,5)P_2_ has been shown to inhibit the activity of this kinase (by approximately 50%) [44]. Noting that PtdIns3P is incorporated into the variable *P*_*p*_ implementation introduces no new parameters, instead [*PIP*2] increases $\left[P_{p}]\to [PI34P_{2}\right]$ (UOM change to $\theta_{4}$). Eqs (1e,1f)
A09	**Inclusion of synthesis of PI(3,4)P_2_ from PtdIns4P.** The synthesis of PI(3,4)P_2_ from PtdIns4P was included in a previous model of the phosphoinositide pathway [10] and the kinase PIK3C2A that has been shown to catalyse this reaction is known to exist in platelets [45]. 1 new parameter (*r*_6_) to represent the rate of transfer from [*PI*] to [*PI*34*P*2]. Eqs (1a, 1e)
A10	**The influence of lysophosphatidylinositol.** Platelets contain phospholipase A02 (cPLA2) [46], an enzyme that can catalyse the hydrolysis of PI (PM) into (LPI) and arachidonic acid (precursor of thromboxane A02) which can be recycled back to PA and PI [47]. This reaction can provide an alternative pathway to slow down the flux of PI to PI4P. Introduce 1 new parameter ($\theta_{-2}$) for the flux from [PI] to [PI4P] that is promoted by IP3 (acting as a surrogate for Ca^2 + ^). Eqs (1a,1i)

### 2.3. Connecting models and data

We are interested in which of the competing hypotheses are important in explaining the experimental data. To test this we use a hybrid approach as described previously [16]. Parameter values for the rate of IP_1_ accumulation from IP_3_ and its transfer to IP are available from literature [48,49], allowing us to set $\theta_{1}∼0.04$ and $r_{3}∼0.0002$ sec^−1^. Prior parameter ranges for all other parameters were set to cover biologically feasible values (10^−4^–10^2^). From these wide ranges parameter values are drawn randomly via a Latin Hypercube and passed to a constrained local optimisation routine (MATLAB’s fmincon) that varies all unknown parameters to minimise the differences between the model structures and experimental data via the cost function

$$\text{Sum Squared due to Error (SSE)}={\left(y_{i}^{j}(\theta )-{\text{Data}}_{i}^{j}\right)}^{2}$$ (3)

where $y_{i}^{j}(\theta )$ is the model’s prediction for the relevant model variable *j* at time points *i* (which depends on the parameters *θ*) and Data${\;}_{i}^{j}$ represents the respective experimental observations. This cost function allows us to define each models ability to fit the data where we mitigate against a more complex model, with more parameters, being able to better fit the data by using Akaike Information Criteria (AICc).

$${\text{AIC}}_{c}=\text{AIC}+\frac{2K(K+1)}{n-K-1}\;\text{where}\;\text{AIC}=n(ln(SSE/n))+2K,$$ (4)

where *K* is the number of parameters and *n* the number of observations. This modified criterion taking into account the experimental sample size by increasing the relative penalty for model complexity with small datasets. The value of AICc has no meaning in isolation, its relevance only becoming apparent when it is used to compare (and rank) models fitted to the same experimental data [50]. Parameter values that enable the model to ’best’ describe the experimental data are saved, the process being repeated 20,000 times for each model. The numerical simulations generated from these parameter values are then compared to assess if they produce biologically consistent and feasible predictions. The ranges of the best-fitting parameter values provide insight into the mechanisms that enable a particular model to describe the data better than alternative models.

### 2.4. Assessing the effect of perturbations

We perform a local sensitivity analysis, where key parameters are varied by 50 percent above and below their initially estimated value, and compute the normalised local sensitivity of the steady state (or endpoint) according to

$$\text{Sensitivity Score}=\frac{O_{a}-O_{i}}{O_{a}},$$ (5)

where *O*_*i*_ and *O*_*a*_ represent the model output (e.g. the steady state achieved in simulations) in respect of the initial parameter set (obtained from parameter fitting) and the adapted parameter respectively.

Simulations are produced that assess the effect of perturbing the pathway via the inhibitor GSK-A1. To simulate this the parameter governing the rate of conversion of PtdIns to PtdIns4P (*r*_1_) is set to 10 percent of its original value and predictions are compared to the experimental data.

Simulations to predict the effect of varying the magnitude and shape of stimulus (*s*(*t*)) have parameters (*a*_1_ = 0.001, *a*_2_ = 0.0002, *a*_3_ = 1.0 and *a*_4_ = 0.02) from Eq 2 adjusted to produce i) a stimulus with a magnitude fifty percent lower than the original used in model calibration (*a*_1_ = 0.001, *a*_2_ = 0.0002, *a*_3_ = 0.5 and *a*_4_ = 0.02); ii) to produce a stimulus with an alternative time-dependent profile, introducing an early transient peak (*a*_1_ = 0.03, *a*_2_ = 0.002, *a*_3_ = 1 and *a*_4_ = 0.02); iii) and to produce a combination with a reduction in magnitude and an early peak (*a*_1_ = 0.03, *a*_2_ = 0.0002, *a*_3_ = 0.5 and *a*_4_ = 0.02).

## 3. Results

### 3.1. Time-dependent experimental data

**Fig 3:**
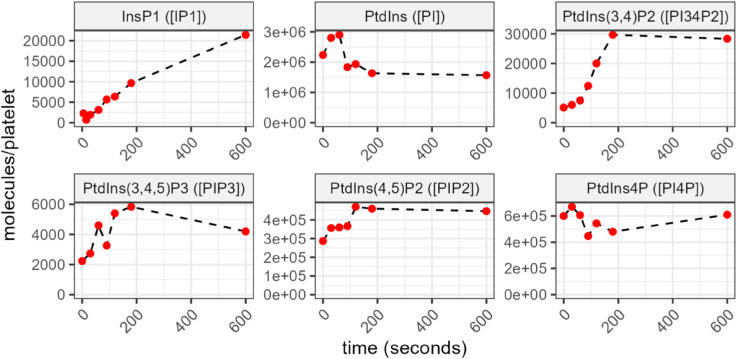
Experimental data showing the time-dependent changes in five phosphoinositides and one inositide. Results are means from 3 experiments. The variable names used in the mathematical models are shown in brackets. See Sect 3.1 for details of data collection.

The experimental data describing the time-dependent changes in five phosphoinositides and the inositol IP1, following stimulation of platelets through the GPVI receptor, are shown in Fig 3. PtdIns, the precursor of all phosphoinositides shows the highest abundance, it rises from 2.3 ± 0.5 to 2.9 ± 0.6 × 10^6^ molecules/platelet over the first 60s before declining to a steady state below basal level (1.5 ± 0.2 × 10^6^ molecules/platelet). The maximal levels of PtdIns4P and PtdIns(4,5)P_2_ are an order of magnitude less than PtdIns. While no significant change was observed for PtdIns4P, its level remaining near the basal, the amount of PtdIns(4,5)P_2_ gradually increased 1.7-fold over the first 120 s (from 2.9 ± 0.6 to $4.8\pm 0.4\times 10^{5}$ molecules/platelet), and remained elevated. PtdIns(3,4)P_2_ shows the greatest change among the phosphoinositides, increasing 6-fold (from 0.5±0.2 to $3.0\pm 0.8\times 10^{4}$ molecules/platelet) over the first 180 s to levels still an order of magnitude lower than PtdIns(4,5)P_2_ with this elevated level being maintained. PtdIns(3,4,5)P_3_ was the least abundant phosphoinositide measured here, a 2.6-fold increase was observed over the first 180 s, from 2.2 ± 0.8 to 5.8 ± 2.8 × 10^3^ molecules/platelet, the large data variance can be attributed to donor variability and/or the low abundance of PtdIns(3,4,5)P_3_ which increases the impact of background noise and lowers the accuracy of the measurement. The inositol IP1 linearly increases to 2.1 × 10^3^ molecules/platelet over 10 minutes, which align with previous reports by Chen et al. who showed CRP-induced accumulation of IP_1_ rising from 200 nM to 600 nM in 2 min (using washed platelets at $8\times 10^{8}$ cells/mL) [51].

Collectively the data emphasise that the inositol phosphoinositides and their metabolites are highly dynamic, reacting rapidly to stimulation on a time scale of seconds to a few minutes. They can be seen to be maintained at levels of abundance that vary by orders of magnitude generally decreasing with each species downstream of PtdIns and this raises questions about how the phosphoinositide pathway acts to maintain these disparate levels.

### 3.2. Model comparisons to data identify the importance of spatial regulation and trafficking

We are interested in which, if any, of the mathematical models, each representing a unique hypothesis concerning the key mechanisms driving the phosphoinositide pathway, are supported by the experimental data. The first step is to determine if a model is able to generate the data via adjustments in the rates of the reactions (parameters). Table 4 provides information on this via the cost function (SSE), which summarises the distance between the experimental data and the models simulations, a lower number reflecting a closer fit to the data. This number is supplemented with the AICc which adjusts the SSE to account for the added complexity induced by adding parameters to the models. Sorting the models in order of their AICc gives


A0∼A01∼A03∼A09<A07∼A04∼A02∼A10<A06<A05≪A10

indicating that the simplest model A0 and three alternative models (A01, A03, A09) can generate the data equally well (AICc in the range of –94 to –90). The complexity of these models varies, with model A09 having one more parameter than A0 and models A03 and A04 having two extra parameters. Models A03 and A04 also add an additional variable, representing cytosolic levels of PI and stabilised PtdIns(3,4,5)P_3_ respectively.

**Table 4:** Results of fitting each model to the data. SSE = distance of model simulations from experimental observations (as defined in the cost function (Eq 3)), n = the number of experimental observations (7 timepoints ×6 datasets  = 42) and K = the number of the model’s parameters that are inferred from the data. Median indicates the median of the lowest 100 results. Metrics in bold denote the models with the lowest SSE or AICc.

Model	A0	A01	A02	A03	A04	A05	A06	A07	A08	A09	A10
Min. SSE/n	**0.054**	**0.054**	0.072	0.048	**0.054**	0.094	0.082	0.063	0.372	0.055	0.068
Median SSE/n	**0.064**	**0.069**	0.093	0.082	**0.069**	0.272	0.11	0.128	0.628	0.072	0.087
Min. AICc	**–94**	**–91**	–82	**–91**	–85	–59	–75	–87	–2	**–90**	–80
K	15	15	15	17	17	17	15	15	15	16	16

**Fig 4:**
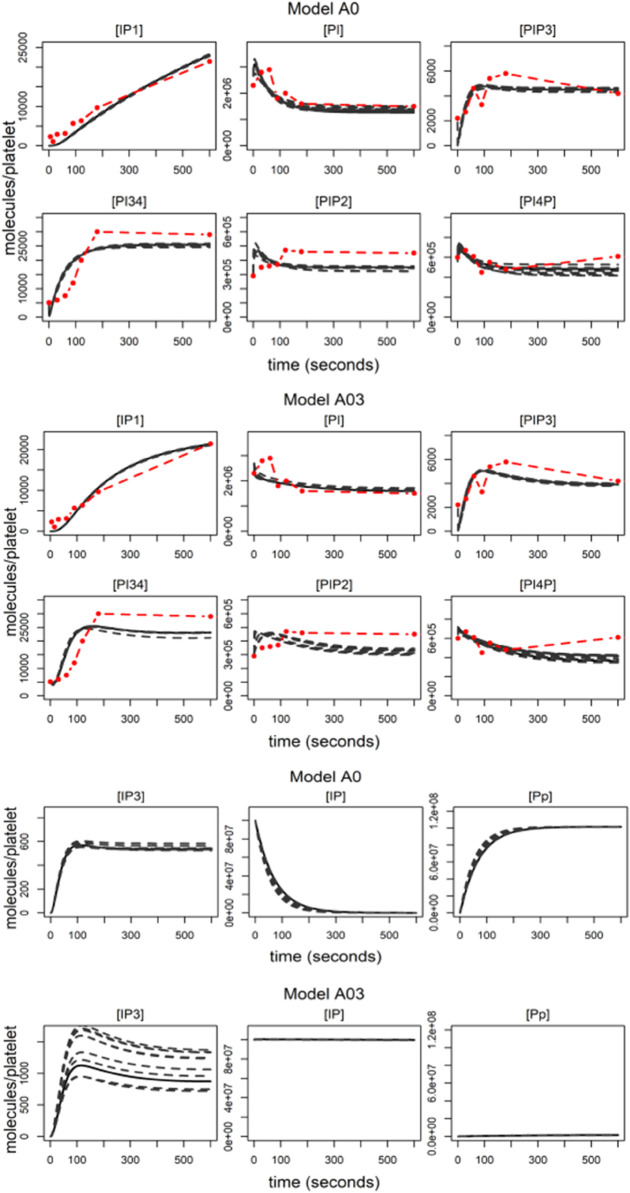
A selection of model simulations. Top two rows shows simulations from model A0 (black lines) compared to data (red), middle two rows show simulations from model A03. Lower panels show simulations of IP3, Ip and Pp for A0 and A03. All simulations show the 10 best fits, the solid line indicating the simulation with the lowest SSE.

Simulations generated from model A0 are shown in Fig 4 (top 2 rows) against the experimental data, those from model A03 are below this (similar simulations for all other models are given in Figs C-H in S1 Text). Here, as in all future plots, we show the simulations generated using the 10 parameters sets with the lowest SSE, demonstrating the uncertainty inherent in inferring complex models from data. Most models are able to produce similar simulations, though models with a higher AICc (A02, A05, A06, A07, and A10) often produce simulations with early, short lived spikes in PtdIns, PtdIns4P and PtdIns(4,5)P_2_. The simulations from model A8 are highly variable and inconsistent with the data reflecting its high AICc.

Three of the models components (variables [*I*_*p*_], [*P*_*p*_] and [*IP*3]) were not measured experimentally. Model predictions for these are shown in the lower two rows of Fig 4. Again, similar predictions generated from the other models are given in Figs C-H in S1 Text. Predictions for changes in the inositol [*IP*3] that is a key step in releasing calcium from internal stores, are generally consistent, showing a sharp rise over the first 100 seconds before settling to a steady state of approximately 600 molecules/platelet. Some models (A03, A05 and A07) have more variability in predictions but are of a similar pattern. Simulations generated by model A08 are highly variable and inconsistent.

Most models predict a quick change in concentration of the soluble pool of inositol (variable [*I*_*p*_]) that drops from 1 × 10^8^ to 0 molecules/platelet in under 300 seconds and there is a related rise in the pool of phosphoinositides ([*P*_*p*_]) rising from 2 × 10^4^ to approximately 1 × 10^8^ (see Model A0 simulations for an example (Fig 4, 5th row)). These predictions seem too drastic to be plausible and the steady state of the variable *P*_*p*_ too high given that it captures the group of phosphoinositides that couldn’t be measured because of their low-abundance. Model A03 is the exception (Fig 4, bottom row), predicting that the pool of inositols is relatively stable with a gradual increase in *P*_*p*_, which is supported by reports of a 3-fold increase in PtdIns3P following CRP stimulation [52]. In all but Model A03 the large pool of soluble inositides is readily available to be reincorporated at the cell membrane to form PtdIns - fueling the phosphoinositide pathway. Model A03 allows a brake to be placed on this process by having some of the pool of PtdIns located in a separate intracellular compartment, this highlights the importance of spatial regulation and trafficking on regulating the cycle and equilibrium of the phosphoinositides.

In summary, four of the models (A0, A01, A03 and A09) are able to simulate the data equally well. But, model A03, that incorporates a simplistic distribution of PtdIns, generates predictions that are more biologically plausible than those from models A0 (a model with no additional regulation), A01 (a model that has regulation in the synthesis of PtdIns4P) and model A09 that introduces synthesis of PtdIns(3,4)P_2_ from PtdIns4P. Model A08, that captures the role of PtdIns 4-kinase in phosphorylating PtdIns3P to PtdIns(3,4)P_2_, is unsupported by this experimental. Noting this, and that implementation of this model was a simplification that may have influenced its ability to simulate the data, model A08 is discarded.

### 3.3. Inferred rates of reactions highlight points of regulation

**Fig 5:**
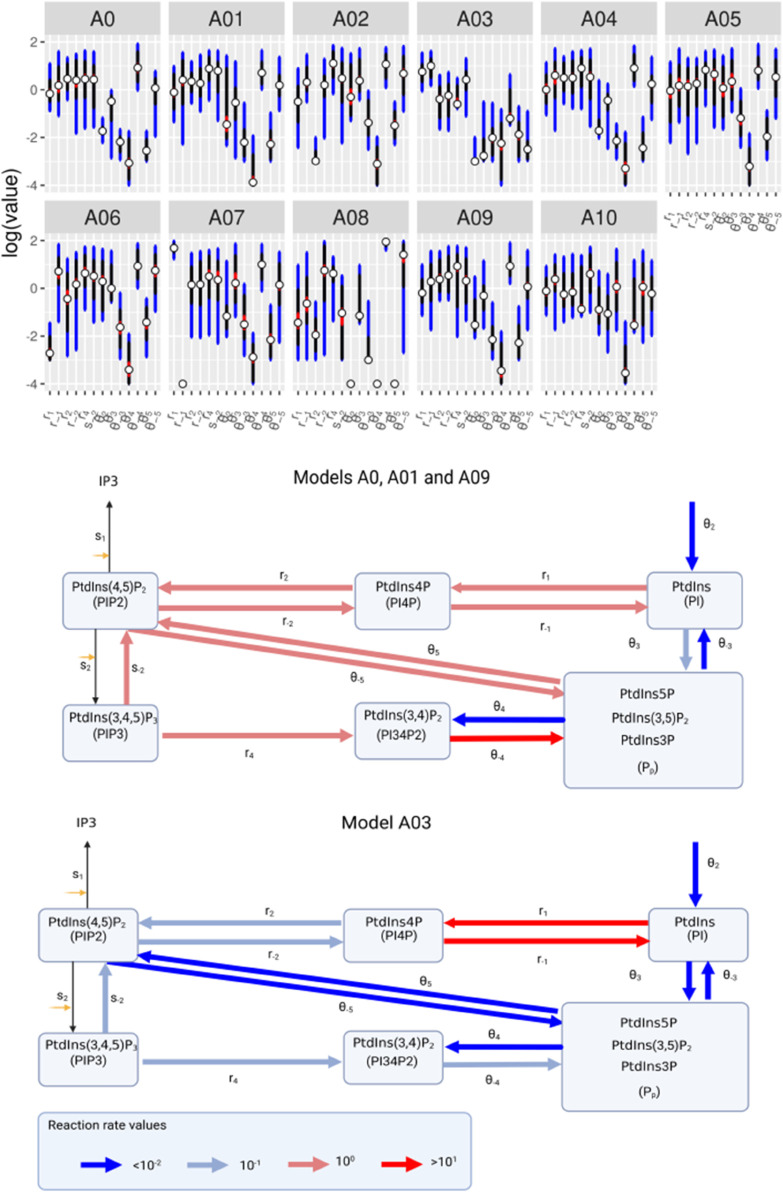
A comparison of the reaction rates inferred by the models. The uncertainty ranges (top) are based on the 100 best (as defined by Eq 3) fits where blue, black and red colours indicate 5 to 95, 25 to 75 and 45 to 55 quantiles respectively and circles represent medians. The prior distributions, from which possible parameter values are drawn, are 10^−4^ to 10^2^ for all parameters. Only parameters common to all eleven models are shown, other posteriors are depicted in S1 Text (Fig B). The lower panels show two schematics of the key reactions around the pathway. These highlight the relative rates of the parameter vales inferred for Models A0, A01, A09 and A03, these being the models best able to fit the data.

The rates of the reactions captured in the models are generally unknown, instead these were inferred from the experimental data. 20,000 possible combinations of reaction rates where sampled from a wide range of values (called priors), with the ’best’ values, those that allowed the model to generate the data (lowest SSE), forming the uncertainty ranges depicted in Fig 5. These sets hold information on which parts of the signalling pathway can be best inferred from the data as well as a parameters influence on the general ability of the model to fit the data, two concepts that are intimately linked. If an uncertainty range is broad and, therefore, not very different from the prior, then the parameter is not inferable from the data and the fitting process is insensitive to its variation. In contrast, parameters with a narrower uncertainty range (lower values, denoted by asterisks, indicate the best constrained) they have had information returned from the experimental data, and the predictions for the data are sensitive to its variation.

Generally parameter uncertanty ranges are well dispersed. Yet, despite this unidentifiability that is present in all of the models some models are better at constraining the data than others. It is worth noting that model A08, that was discarded for its inability to fit the data, has wide uncertainty ranges for most parameters in combination with parameters at identified to be at the extremes of the priors for some reactions. This is particularly true for parameters, such as $\theta_{4}$, that counteract A08’s newly introduced mechanism that promotes the rate that the unmeasured phosphoinosities ([*P*_*p*_]) are converted to PtdIns(3,4)P_2_. Models A0, A01 and A09 (and indeed many others) infer fast rates around the pathway and into the pool of unmeasured phosphoinosities, with rates out of the pool generally 4 orders of magnitude slower. Model A03 constrains the reactions rates differently, having slower rates into the pool. The differences in rates inferred by models A0, A01, and A09 compared to A03 are evident in their simulations, with the former showing increases in the pool of unmeasured phosphoinositides. This variation in rates between the models is further illustrated in the schematics in the lower panels of Fig 5, where red arrows indicate faster reaction rates and blue arrows denote slower ones. Specifically, the reactions governing the progression from [PI] through [PI4P], [PIP2], [PIP3], and [PI34P2] to [P_*p*_] (*r*_1_, *r*_−1_, *r*_2_, *r*_4_, $\theta_{-4}$) fall within the range of 0.1–10 per second, significantly faster than those observed in model A03.

In summary, most models that fit the data well predict a fast flow through the phosphoinositide pathway to the unconstrained pool of unmeasured phosphoinositides, this facilitating model fits to data. This is not the case for model A03. Here, the concentration of PtdIns, the precursor of the other phosphoinositides, is reduced at the membrane. The newly introduced cytosolic of PtdIns being protected from rapid incorporation. This provides a break on the pathway and limits the fast early spikes often seen in simulations from other models and increases in the pool of unmeasured phosphoinositides.

### 3.4. Model predictions under the effect of inhibitors and perturbations

Alterations in phosphoinositide metabolism have been found to underlie many disease states, this being supported by the discovery of mutations in the kinases that regulate the pathway that lead to disease progression [53]. To perform model validation, experimental data was generated under the effect of the PI4KA inhibitor (GSK-A1) that blocks conversion from PtdIns [54], reducing levels of PtdIns4P.

**Fig 6:**
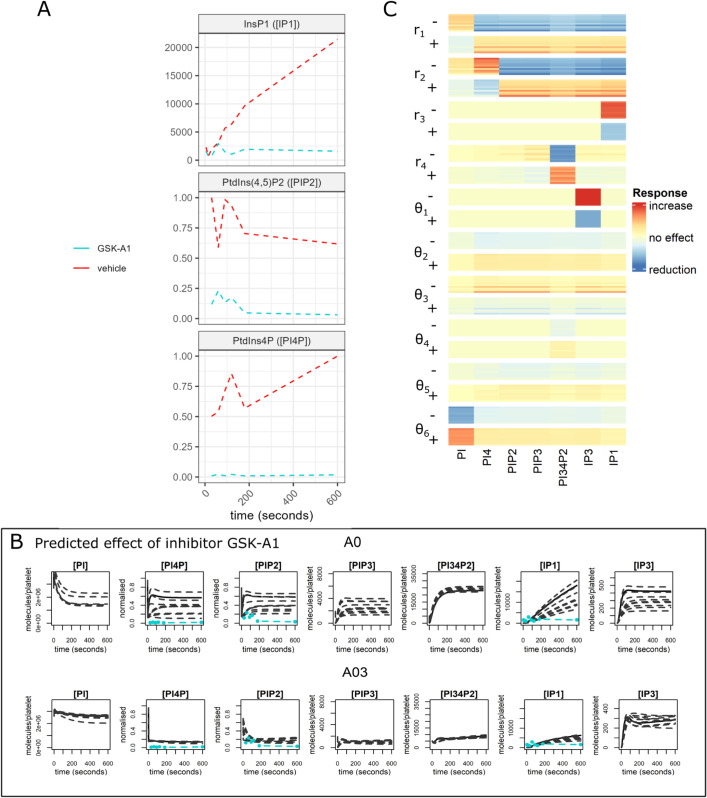
The effects of inhibitors and perturbations.. A) Experimental data demonstrating the effect of the inhibitor GSK-A1 on IP1 accumulation, PtdIns4P and PtdIns(4,5)P_2_. Results without inhibitor shown in red, treated with GSK-1 in cyan. B) A comparison of predictions for the effect of the inhibitor GSK-A1 from models A0 and A03. Simulations (black) are from the 10 best fits, the solid line indicating the simulation with the lowest SSE. Experimental data is in cyan. C) Heatmap demonstrating the results of a local sensitivity analysis on the 10 best fits from model A03. The heatmap shows the effect of varying key parameters (by 50% up (+) and down (-)) on model outputs. Red indicates a positive effect, blue a negative effect. The intensity of the colour corresponding to the magnitude of the effect.

Simulations of all models were run using the 10 parameter sets with lowest SSE while adjusting the parameter governing the rate of conversion of PtdIns to PtdIns4P (*r*_1_). In-vitro experimental data is shown in Fig 6, A where it can be seen that treatment with GSK-A1 greatly reduced CRP-induced IP_1_, PtdIns4P and PtdInss(4,5)P_2_ production so that they remained at near basal level for 10 minutes. Model predictions for the effect of the inhibitor are shown in Fig 6, B (outputs for models A0 and A03 are shown, others are provided in Fig I-N in S1 Text). Model A03 is the only model to consistently have predictions that agree with the new data, predicting only small increases in the accumulation of IP_1_ and rapid decreases in PtdIns4P and PtdIns(4,5)P_2_ from their initial conditions so that they align with the data. Model A03 also predicts large reductions in PtdIns(3,4,5)P_3_ and PtdIns(3,4)P_2_ (to approximately 20% of their original level) with IP_3_ accumulation reduced to 30 of that achieved in simulations without the inhibitor. This is accompanied by slight increases in PtdIns, reflecting its lack of conversion to PtdIns4P and its downstream phosphoinositides. Most models other than A03 produce variable predictions for many species, but generally models that show a reduction in IP_1_ also predict a reduction in IP_3_.

We have seen above that varying the rate of a single parameter (*r*_1_) can effect the whole posphoinositide pathway. The effects of varying other parameters are investigated via a local sensitivity analysis (of model A03). The results are presented in the heatmap (Fig 6, C) where, for simplicity, only the effects of varying forward reactions are shown, each varied by fifty percent above and below their original value. As we saw *r*_1_ affects the whole pathway and *r*_2_, the rate of synthesis of PtdIns(4,5)P_2_ from PtdIns4P, can be seen to have a similar effect, although PtdIns4P ([PI4P]) is now increasing. Other parameters have a less global effect. The parameter that governs the degradation of IP_1_ so it can be reincorporated at the membrane (*r*_3_) predictably effects IP_1_ accumulation and *θ*_1_, the rate of transfer from IP_3_ to IP_1_ effects mainly IP_3_ levels. The parameter which controls the rate of conversion from PtdIns(3,4,5)P_3_ to PtdIns(3,4)P_2_ (*r*_4_) has more effect on the latter, reflecting their relative abundance’s. A03’s the newly introduced parameter ($\theta_{6}$) that controls the transfer from the inositols to the secondary pool of PtdIns, only effects levels of PtdIns and is not passed through to the other phosphoinositides. Variation in parameters $\theta_{3}$, $\theta_{4}$ and $\theta_{5}$ show little effect, reflecting their inability to be inferred from the experimental data.

In summary model A03 is the only model to successfully predict the effect of the GSK-A1 inhibitor. The effects of varying other parameters on the outcomes generated from model A03 show that the model’s outputs are most sensitive to the rate of conversion from PtdIns, through PtdIns4P, to PtdIns(4,5)P_2_.

### 3.5. Phosphoinositide pathway responses under changing receptor stimulation

**Fig 7:**
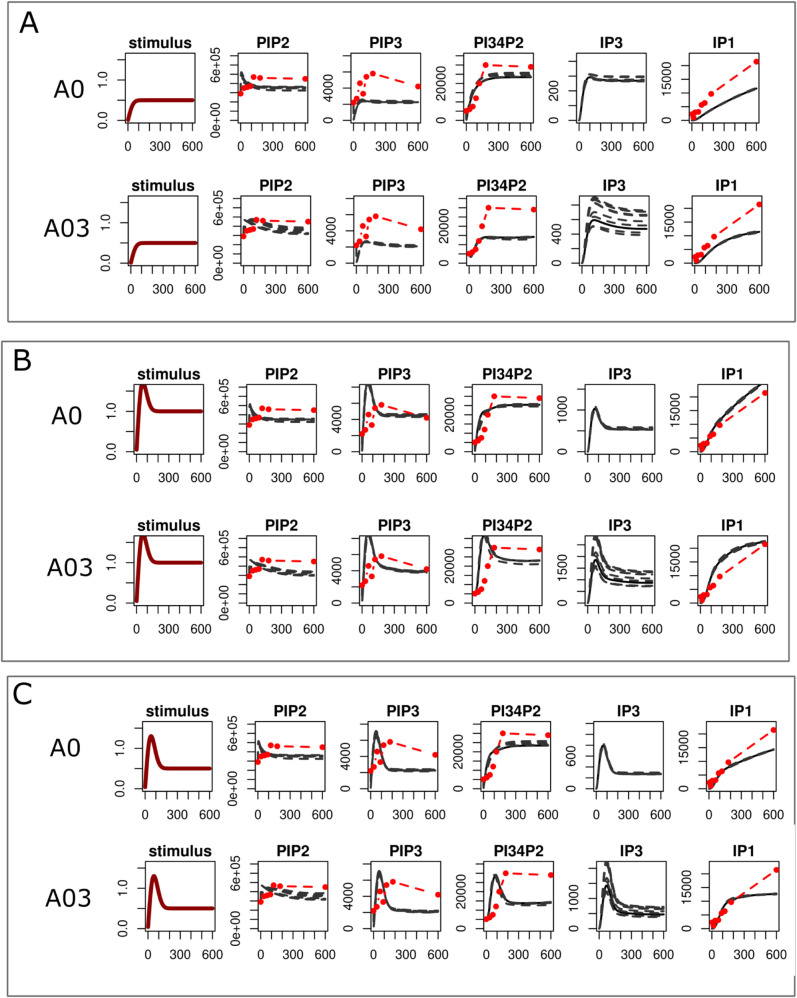
Model predictions under changing levels of stimulus. Simulations shown are predictions generated from two models (A0 and A03), others are provided in S1 Text, to the levels of stimulus. A) a stimulus with a magnitude fifty percent lower than the original used model calibration. B) a stimulus with an alternative time-dependent profile, introducing an early transient peak. C) a combination of the stimulus for the simulations in panels A and B introducing an early peak and a reduction in the steady state.

In our models the stimuli (Eq 2) promotes hydrolysis of PtdIns(4,5)P_2_ to IP_3_ and its catalysis to PtdIns(3,4,5)P_3_. Fig 7 provides three examples of changes in the magnitude and shape of this stimuli, and the effect they have on model simulations (others are provided in Figs O-Q in S1 Text). In the first example *s*(*t*) is adjusted so that there is a 50% drop in magnitude from that used for model calibration, the second has an alternative non-linear time-dependent profile characterised by an early transient peak and the third combines the two effects having an early transient peak before settling to a lower steady state.

Model predictions to these changing stimuli are generally consistent. They show that stimuli affects the magnitude and the dynamics of PtdIns(3,4,5)P_3_, IP_3_ and IP_1_ but not PtdIns(4,5)P_2_. A 50% reduction in the magnitude of the stimulus leads to a similar drop in the magnitude of PtdIns(3,4,5)P_3_, IP_3_ and IP_1_ and the introduction of an early peak in the stimulus was replicated in the profiles of PtdIns(3,4,5)P_3_ and IP_3_ but not IP_1_ where accumulation has a monotonic profile that saturated at later time points. There are variations to these predictions, especially in PtdIns(3,4)P_2_ which is predicted to drop and/or display an early peak in simulations from models A0, A01, A02, A03, A04 and A07.

Ligand specific regulation of signalling events in platelets (or indeed other cell types) are thought to alter phosphoinositides composition [3]. The mathematical models developed here offer a way of exploring how differing cellular activities may alter the phosphoinositides pathway. We find that while signals are generally quickly propagated through the phosphoinositides, PtdIns(4,5)P_2_ remains remarkably robust in the face of changing stimuli.

## 4. Discussion

The study of phosphoinositides brings with it major challenges, not least is their measurement that is made difficult by their low abundance and high polarity [1,9]. Here we have utilised a new dataset that describes the time-dependent changes of five phosphoinositides and an inositol in platelets following stimulation with a single ligand, CRP. To extract the most information from this unusually dense dataset we have used mathematical and computational techniques that allow us to test our ideas on how the phosphoinositide pathway is regulated against the data. Mathematical models of such complex biological systems combine uncertainty in their structure, there often being competing ideas about how molecules interact, and in the rates of these reactions. We constructed eleven alternative representations of the pathway and use a computational technique that alleviates the need to base reaction rates on literature where they would necessarily have been measured in other cell types and experimental conditions, to generate simulations. This framework allows us to compare and assess which model structures are supported by the data while at the same time inferring rates of reactions and quantifying uncertainty in their and the models predictions.

Our models capture and test many different methods of regulation, both those that are known to exist in platelets and those found to play a role in regulating the phosphoinositides in other cell types. We found that while all but one of the models was able to generate the experimental data only a model with a secondary pool of PtdIns (model A03) was able to provide realistic predictions. The secondary pool acted as a brake on the availability of PtdIns, slowing its conversion to the other phosphoinositides. It was this model that resulted in realistic predictions that compared favourably to those from other models that predicted a sharp decline in the inositols and a respective rise in phosphoinositides, the later being known to occur in low abundance. Model A03 was also able to successfully generate predictions in line with the data under the influence of the inhibitor GSK-A1. It has long been known that PtdIns(4,5)P_2_ has a high turnover rate, which has been linked to the so-called futile cycles of dephosphorylation and rephosphorylation that are thought to occur on the plasma membrane [55]. This is demonstrated by the rapid labelling kinetics of PtdIns(4,5)P_2_ and PtdIns4P that is in contrast to the slower labelling kinetics of PtdIns and other phospholipids [56]. Controlling the recycling of the phospholipids such as PtdIns(4,5)P_2_ that make up only a tiny proportion of all cellular PtdIns is crucial for regulating signalling and membrane dynamics [57]. Our investigation into the response to changing levels and shape of stimulation to the pathway found that these differentially affected phosphoinositide species. With a 50% drop in the magnitude of the stimulating signalling events the phosphoinositides PtdIns(4,5)P_2_ and PtdIns(3,4,5)P_3_ dropped by a similar amount. But, in agreement with Hille and coworkers [13], PtdIns(4,5)P_2_ levels were robust in the face of changing levels of stimulation, not being depleted. It was only by disrupting the rates of internal phosphoinositide conversion (with the inhibitor GSK-A1) that levels of PtdIns(4,5)P_2_ could be reduced.

The models presented here represent a necessary simplification of a process that is evidently more complex, particularly in the spatial events that underpin and regulate the availability of individual lipid pools [34,57]. In our models, spatial events have been incorporated in a rudimentary manner, as exemplified by model A03, where a secondary pool of PtdIns is introduced to limit the amount of inositol available at the cell membrane. Developing and testing models that include a more detailed representation of spatial localisation would require either a better understanding of the reaction rates between spatial locations or localised experimental data, neither of which is currently available.

In our present models, the general lack of knowledge regarding the rates of conversion between various species is reflected in the broad priors used when comparing model simulations to data. This results in large uncertainties in parameter estimates that span several orders of magnitude, demonstrating the insufficiency of experimental data to constrain parameter values effectively. It is crucial to remember that no model is entirely accurate or fully identifiable, as the identifiability of parameter values heavily depends on the model structure. We have deliberately kept our models as simple as possible, for example aggregating low abundant species PtdIns5P, PtdIns(3,5)P2 and PtdIns3P into a single variable and this aids our ability to optimise models to the available data. Also, while it is biologically plausible that combinations of our ten regulatory regulatory mechanisms—or indeed alternatives—could play a role in regulating the phosphoinositide pathway, there is currently insufficient data to support such complexity. Consequently, these models should be regarded as incremental steps towards a better understanding of phosphoinositide signalling, which is why we publish the accompanying code to enable researchers to modify and extend these models.

The insights gained from validating the models against data obtained under the influence of inhibitors, as well as the differential responses predicted under varying levels of stimulation, suggest that such additional data could be highly informative in constraining the range of approximated parameters. This approach represents a more achievable goal than attempting to measure other phosphoinositide species (PtdIns3P, PtdIns5P, and PtdIns(3,5)P_2_), where experimental challenges are compounded by their low abundance.

The availability of code to run these models, provided in a widely used programming language among biologists, ensures that researchers can readily adapt the models for their own studies and test them against experimental data. While advances in mass spectrometry-based profiling and imaging techniques have significantly improved our ability to study cellular processes, our quantitative understanding of transient and unstable phosphoinositides remains limited [1]. Nevertheless, our approach of using mathematical models to formalise hypotheses about the regulation of phosphoinositides, combined with a computational framework that allows testing these ideas against a new, experimentally consistent dataset, offers valuable insights. This work provides a step forward in understanding how the delicate balance between phosphoinositide levels is maintained and regulated in platelets, paving the way for further research in this field.

## Supporting information

S1 Text S1 Text includes the equations detailing the changes to model A0 incorporated into models A01–A10 and additional figures. (PDF)
