# Supplementary material for: A computational framework for the investigation of phosphoinositide regulation
Source: PLoS Comput Biol. 2025 Sep 30;21(9):e1013477. doi: 10.1371/journal.pcbi.1013477 (PMC12510658; doi:10.1371/journal.pcbi.1013477)
Supplement: S1 Text — (PDF) [file pcbi.1013477.s001.pdf]

A COMPUTATIONAL FRAMEWORK FOR THE INVESTIGATION OF PHOSPHOINOSITIDE REGULATION - SUPPLEMENTAL.

A PREPRINT

Yam Fung Hilaire Cheung[2,3,4], Chukiat Tantiwong[1], Dipali Kale[4], Jonathan M Gibbins[1], Steve Watson[2], Johan W.M. Heemskerk[3], Albert Sickmann[4], Robert Ahrends[5] and Joanne L. Dunster[1]

[1]Institute for Cardiovascular and Metabolic Research, School of Biological Sciences, University of Reading, Reading, UK
[2]Department of Cardiovascular Sciences, College of Medicine and Health, University of Birmingham, Birmingham, UK
[3]Department of Biochemistry, Cardiovascular Research Institute Maastricht (CARIM), Maastricht University, Maastricht, The Netherlands
[4]Leibniz-Institut für Analytische Wissenschaften-ISAS-e.V, Dortmund, Germany
[5]Dept. of Analytical Chemistry, University of Vienna, Vienna, Austria

September 23, 2025

In Section S1 we present the equations detailing the changes to model A0 incorporated into models A01-A10. Fig A provides details of the data and fitting of the function $s(t)$. Fig B provides uncertainty ranges of parameter values to supplement those of Fig 5, main text. Figs C, D, E, F, G and H show simulations from all eleven models. Figs I, J, K, L, M and N show predictions from the models under the influence of the inhibitor GSK-A1 and Figs O, P and Q show predictions for varying the stimulus.

S1.1 Modifications to model A0.

Model A01 introduces no new parameters. Stimulation (through the function $s(t)$ now promotes r_4, modifying equations (1d) and (1e) so that

$$\frac{d[PIP3]}{dt} = s_2 s(t)\,[PIP2] - s_{-2}\,PIP3 - r_4\,s(t)[PIP3],$$
$$\frac{d[PI34P2]}{dt} = r_4 s(t)[PIP3] + \theta_4 P_p - \theta_{-4}\,[PI34P2].$$

Model A02 introduces no new parameters, it has variable IP3 promoting r_2 so that equations (1b) and (1c) now read

$$\frac{d[PI4P]}{dt} = r_1\,[PI] - r_{-1}[PI4P] - r_2\,[PI4P]\,[IP3] + r_{-2}[PIP2],$$
$$\frac{d[PIP2]}{dt} = r_2\,[PI4P]\,[IP3] - r_{-2}[PIP2] - s_2 s(t)\,[PIP2] + s_{-2}\,[PIP3] - s_1 s(t)[PIP2] + \theta_5[P_p] - \theta_{-5}[PIP2].$$

Model A03 incorporates 2 new terms into equation (1i) so that

$$\frac{d[I_p]}{dt} = r_3[IP1] - \theta_6[IP1] + \theta_{-6}[PI_c],$$

and a new equation

$$\frac{d[PI_c]}{dt} = \theta_6[IP] - \theta_{-6}[PI_c] - \theta_2[PI_c].$$

Model A04 introduces 2 new terms to equation (1d), such that

$$\frac{d[PIP3]}{dt} = s_2 s(t)\,[PIP2] - s_{-2}\,[PIP3] - r_4\,[PIP3] - s_4\,s(t)\,[PIP3] + s_{-4}\,[PIP3b],$$

and a new equation for the variable [PIP3b]

$$\frac{d[PIP3b]}{dt} = s_4 s(t)[PIP3] - s_{-4}\,[PIP3b].$$

Model A05 introduces 2 new terms to equation (1b), such that

$$\frac{d[PIP2]}{dt} = r_2\,[PI4P] - r_{-2}\,[PIP2] - s_2 s(t)\,[PIP2] + s_{-2}\,[PIP3] - s_1 s(t)\,[PIP2] + \theta_5[P_p] - \theta_{-5}\,[PIP2] \\ -s_5\,[PIP2] + s_{-5}[IP3]\,[PIP2b],$$

and a new equation for the variable [PIP2b]

$$\frac{d[PIP2b]}{dt} = s_5\,[PIP2] - s_{-5}\,[IP3]\,[PIP2b].$$

Model A06 introduces no new parameters, it has variable [IP3] promoting r_1 so that equations (1a) and (1b) now read

$$\frac{d[PI]}{dt} = \theta_2\,[I_p] - r_1\,[IP3]\,[PI] + r_{-1}[PI4P] - \theta_3\,[PI] + \theta_{-3}\,[P_p],$$
$$\frac{d[PI4P]}{dt} = r_1\,[IP3]\,[PI] - r_{-1}[PI4P] - r_2\,[PI4P] + r_{-2}\,[PIP2].$$

Model A07 introduces no new parameters, it has variable PI4 promoting r_{-1} so that equations (1a) and (1b) now read

$$\frac{d[PI]}{dt} = \theta_2\,[I_p] - r_1\,[PI] + r_{-1}\,[PI4P]\,[PI4P] - \theta_3\,[PI] + \theta_{-3}\,[P_p],$$
$$\frac{d[PI4P]}{dt} = r_1\,[PI] - r_{-1}\,[PI4P]\,[PI4P] - r_2\,[PI4P] + r_{-2}\,[PIP2].$$

Model A08 introduces no new parameters, it has variable PIP2 increasing θ_4 so that equations (1e) and (1f) now read PIP2 increases Pp to PI34P2

$$\frac{d[PI34P2]}{dt} = r_4\,[PIP3] + \theta_4[P_p]\,[PIP2] - \theta_{-4}\,[PI34P2],$$
$$\frac{d[P_p]}{dt} = \theta_3\,[PI] - \theta_{-3}\,[P_p] - \theta_5[P_p] + \theta_{-5}\,[PIP2] - \theta_4[P_p]\,[PIP2] + \theta_{-4}\,[PI34P2].$$

Model A9 introduces the term with parameter r_6 to equations (1a) and (1e) such that

$$\frac{d[PI]}{dt} = \theta_2\,[I_p] - r_1\,[PI] + r_{-1}[PI4P] - \theta_3\,[PI] + \theta_{-3}\,[P_p] - r_6\,[PI],$$
$$\frac{d[PI34P2]}{dt} = r_4\,[PIP3] + \theta_4[P_p] - \theta_{-4}\,[PI34P2] + r_6\,[PI].$$

Model A10 introduces the term with parameter θ_{-2} to equation (1a) and (1i) such that

$$\frac{d[PI]}{dt} = \theta_2\,[I_p] - r_1\,[PI] + r_{-1}[PI4P] - \theta_3\,[PI] + \theta_{-3}\,[P_p] - \theta_{-2}\,[IP3]\,[PI],$$
$$\frac{d[I_p]}{dt} = r_3[IP1] - \theta_2\,[I_p] + \theta_{-2}\,[IP3]\,[PI].$$

S1.2 Additional figures.

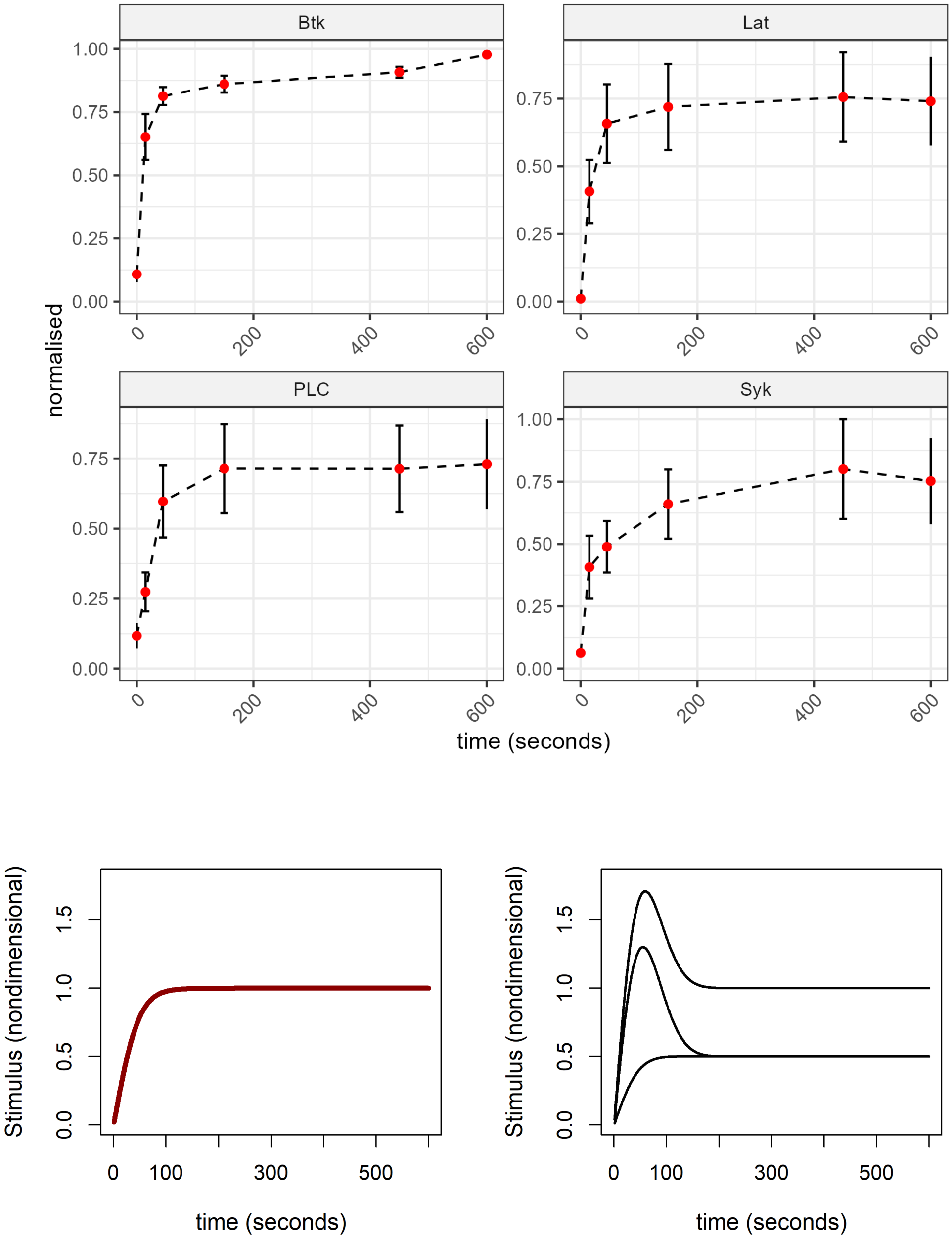

Figure A: Function $s(t)$ compared to experimental data. The top row shows experimental data (median n=4, normalised to maximal responses) describing the phosphorylation of upstream events that occur following platelet activation through the GPVI receptor. This can be compared to the simulations of $s(t)$ (bottom row, left, in red), where $a_1 = 0.001$, $a_2 = 1$ have been adjusted to capture the qualitative shape of data. Simulations of $s(t)$ are also shown (bottom row, left, in black) for $s(t)$ with $a_1 = 0.03, a_2 = 1$; $a_1 = 0.001, a_2 = 0.5$ and $a_1 = 0.03, a_2 = 0.5$, these being used in simulations shown in Fig 7 (main text).

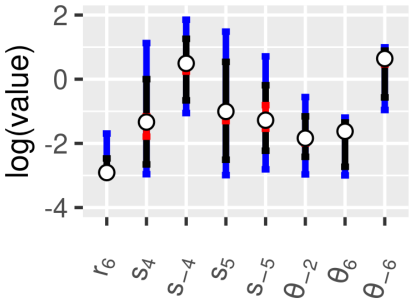

Figure B: Distributions of inferred parameter values for parameters unique to alternate models. These uncertainty ranges are based on the 100 best fits. Blue, black and red colours indicating 5 to 95, 25 to 75 and 45 to 55 quantiles respectively. Circles represent medians. The prior distributions, from which possible parameter values are drawn, are 10^{-4} to 10^2. Parameters θ_6 and θ_{-6} are unique to model A03; s_4 and s_{-4} to model A04; s_5 and s_{-5} to model A05; r_6 to model A09 and θ_{-2} to model A10.

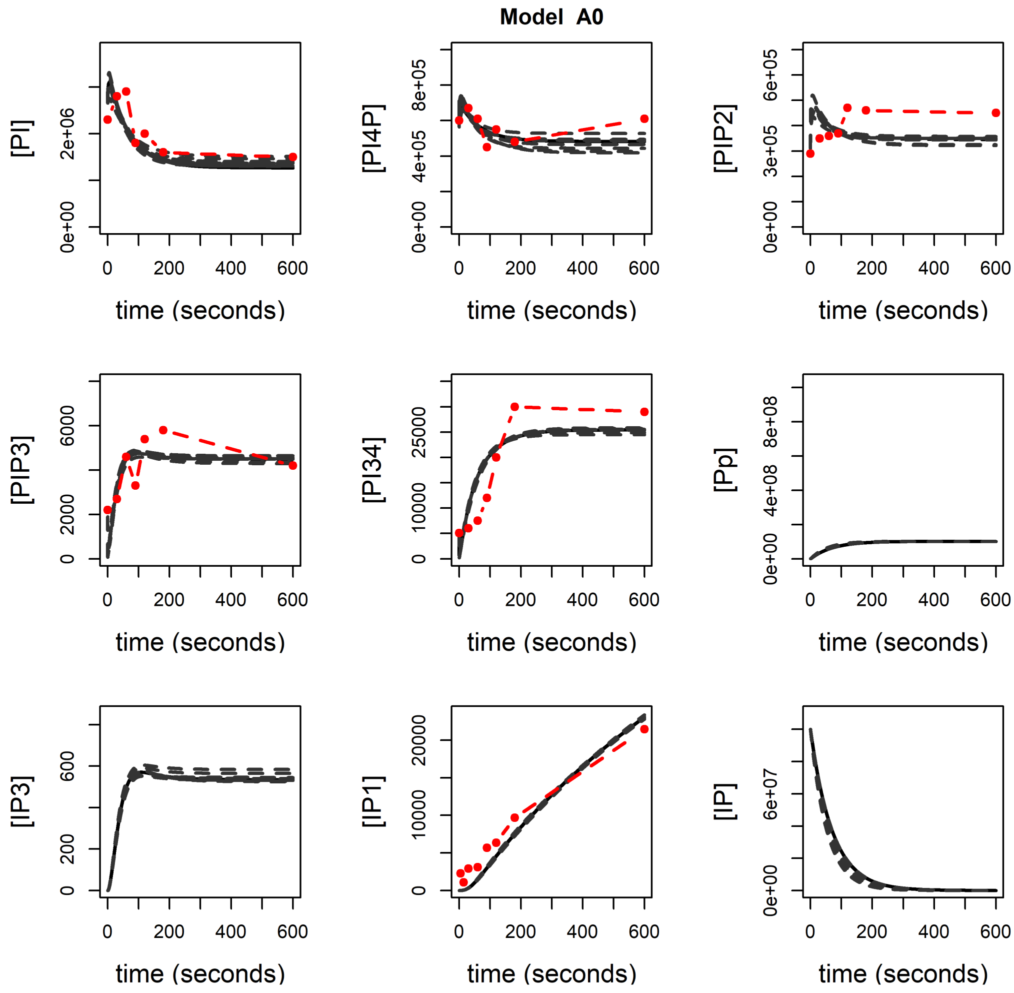

Figure C: Model A0 simulations (black) compared to experimental data (red). Simulations are based on the parameter values from the 10 'best' fits. Solid line denotes simulation with lowest cost function.

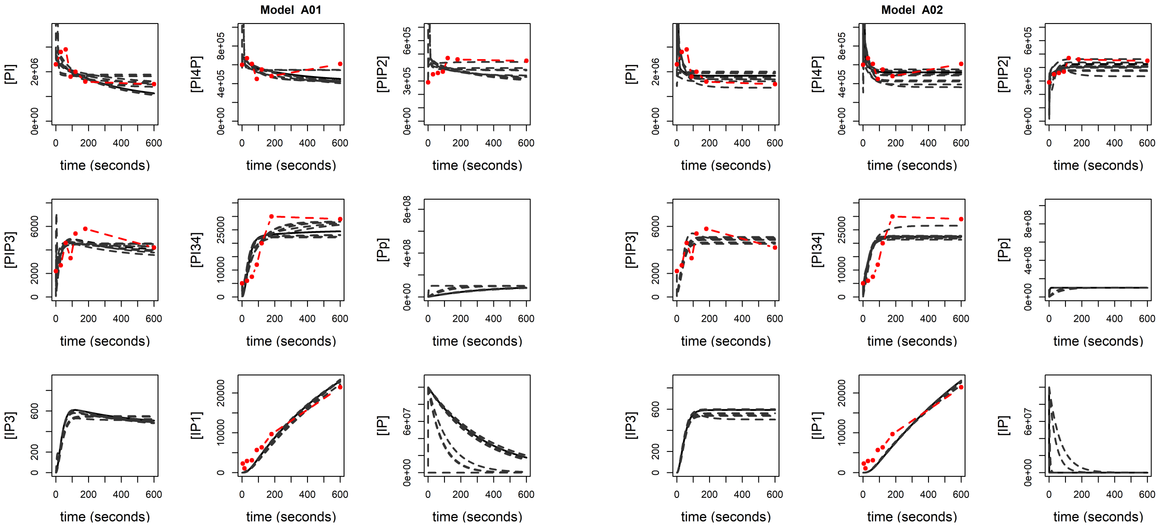

Figure D: Model A01 (left) and A02 (right) simulations (black) compared to experimental data (red). Simulations are based on the parameter values from the 10 'best' fits. Solid line denotes simulation with lowest cost function.

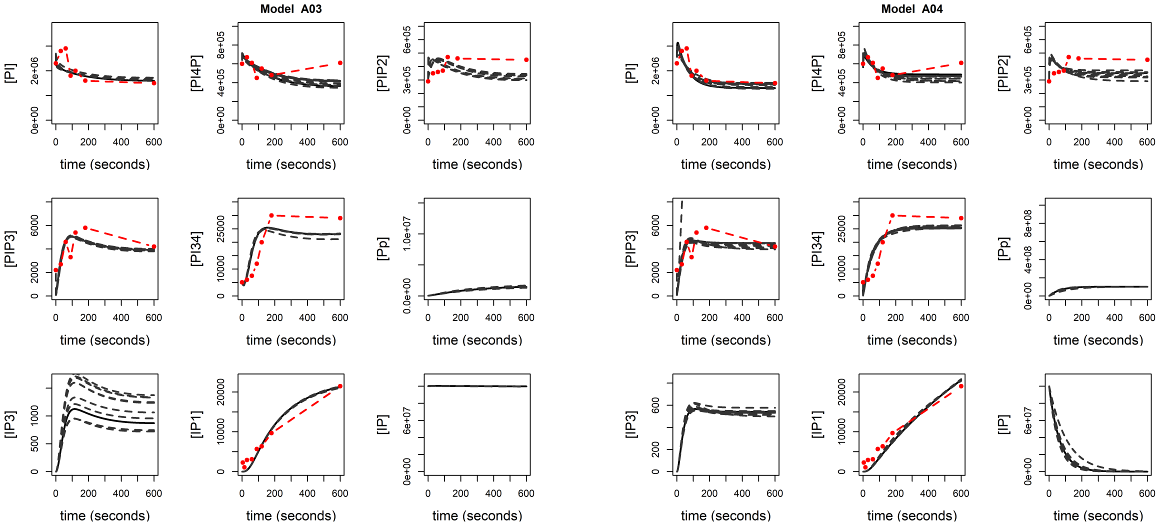

Figure E: Model A03 (left) and A04 (right) simulations (black) compared to experimental data (red). Simulations are based on the parameter values from the 10 'best' fits. Solid line denotes simulation with lowest cost function.

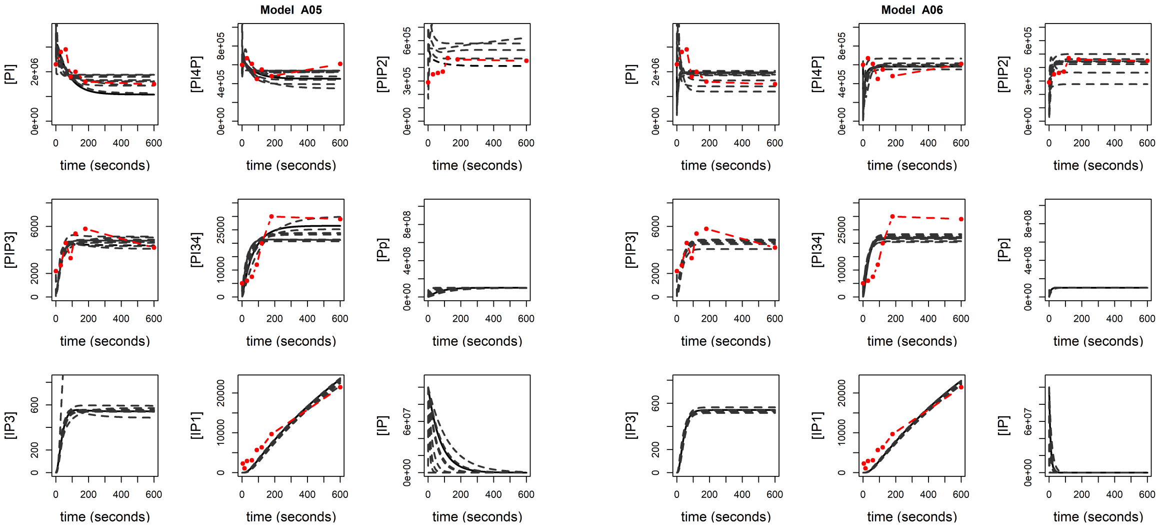

Figure F: Model A05 (left) and A06 (right) simulations (black) compared to experimental data (red). Simulations are based on the parameter values from the 10 'best' fits. Solid line denotes simulation with lowest cost function.

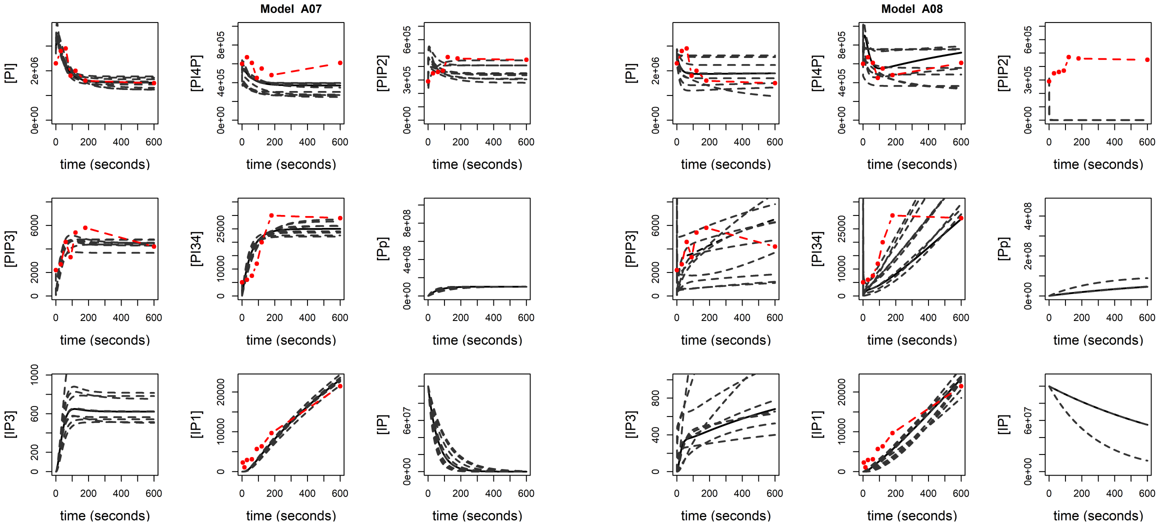

Figure G: Model A07 (left) and A08 (right) simulations (black) compared to experimental data (red). Simulations are based on the parameter values from the 10 'best' fits. Solid line denotes simulation with lowest cost function.

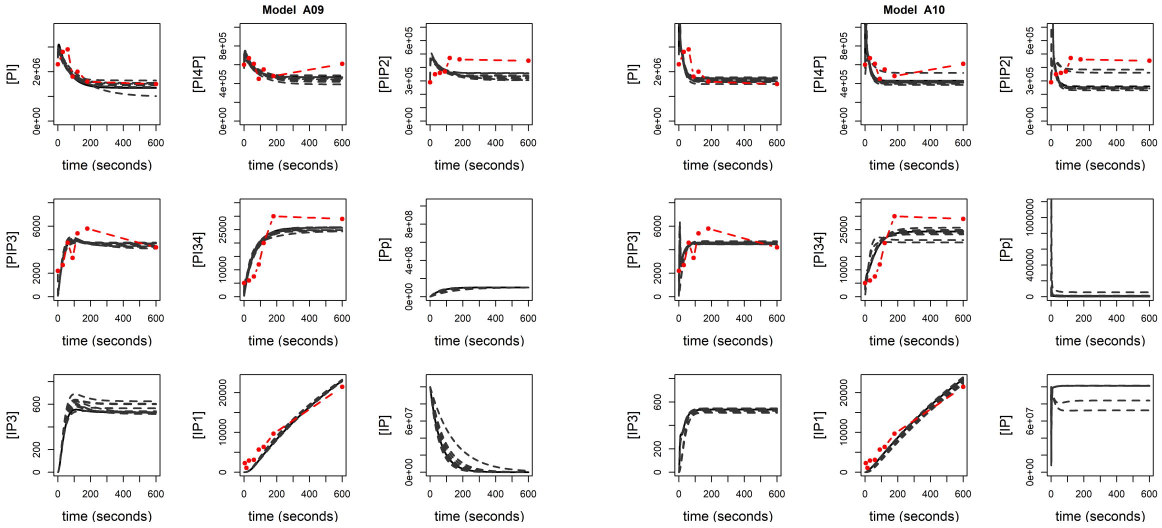

Figure H: Model A09 (left) and A10 (right) simulations (black) compared to experimental data (red). Simulations are based on the parameter values from the 10 'best' fits. Solid line denotes simulation with lowest cost function.

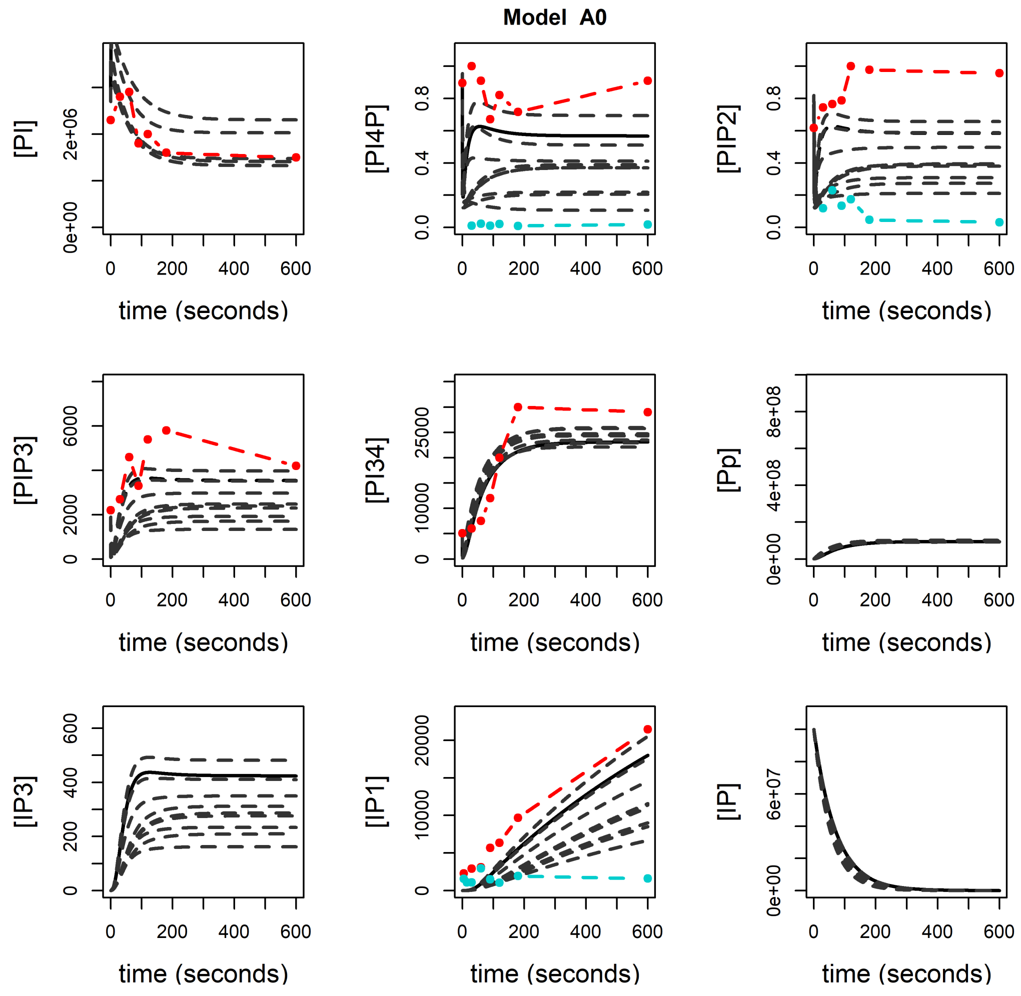

Figure I: Model A0 predictions (black) for the effect of the inhibitor Gsk-A1, compared to the original experimental data (bright red) and data under the effect of the inhibitor (cyan). Simulations are based on the parameter values from the 10 'best' fits. Solid line denotes simulation with lowest cost function.

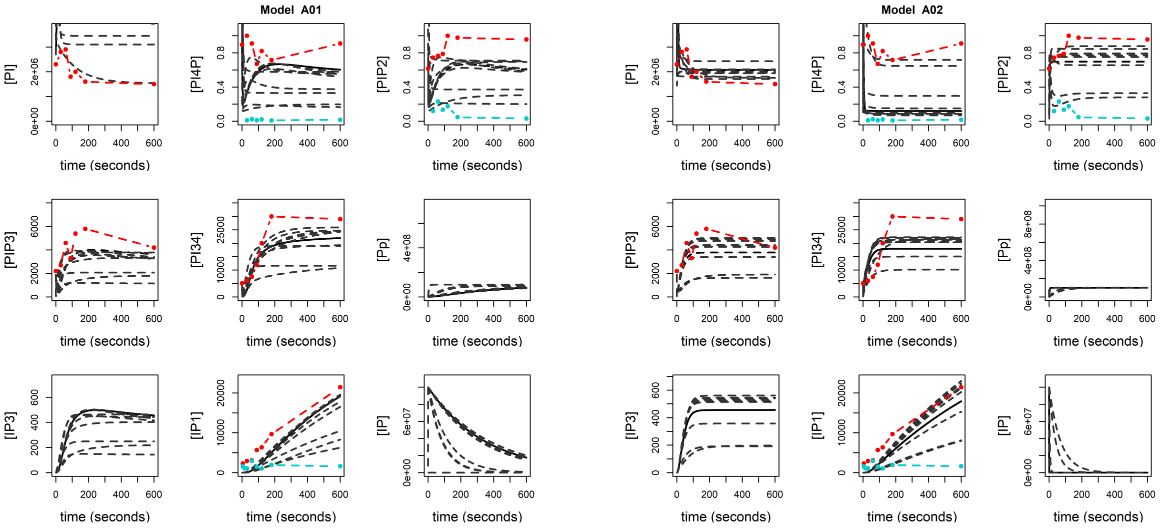

Figure J: Model A01 (left) and A02 (right) predictions (black) for the effect of the inhibitor Gsk-A1, compared to the original experimental data (bright red) and data under the effect of the inhibitor (cyan). Simulations are based on the parameter values from the 10 'best' fits. Solid line denotes simulation with lowest cost function.

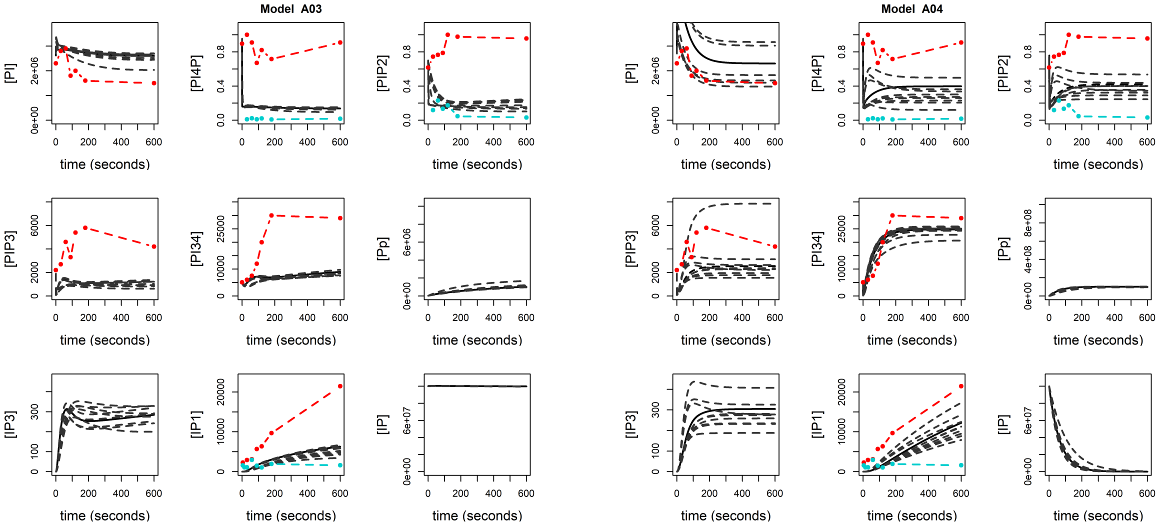

Figure K: Model A03 (left) and A04 (right) predictions (black) for the effect of the inhibitor Gsk-A1, compared to the original experimental data (bright red) and data under the effect of the inhibitor (cyan). Simulations are based on the parameter values from the 10 'best' fits. Solid line denotes simulation with lowest cost function.

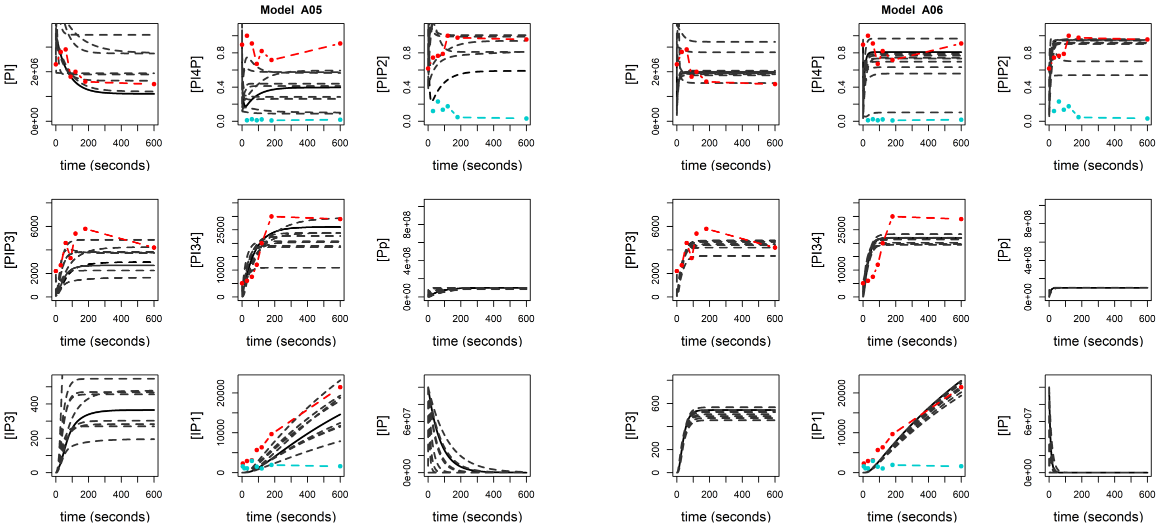

Figure L: Model A05 (left) and A06 (right) predictions (black) for the effect of the inhibitor Gsk-A1, compared to the original experimental data (bright red) and data under the effect of the inhibitor (cyan). Simulations are based on the parameter values from the 10 'best' fits. Solid line denotes simulation with lowest cost function.

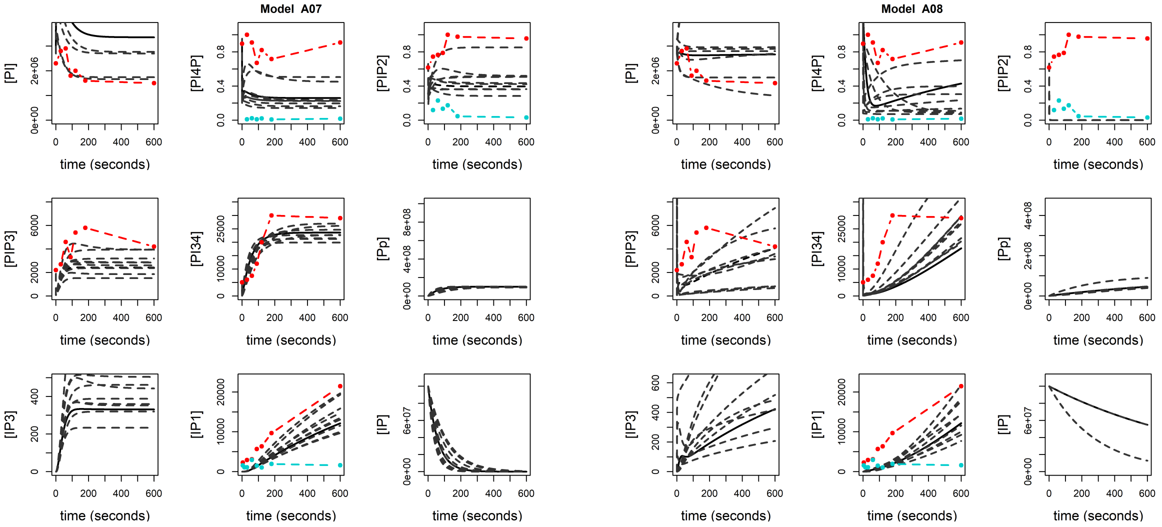

Figure M: Model A07 (left) and A08 (right) predictions (black) for the effect of the inhibitor Gsk-A1, compared to the original experimental data (bright red) and data under the effect of the inhibitor (cyan). Simulations are based on the parameter values from the 10 'best' fits. Solid line denotes simulation with lowest cost function.

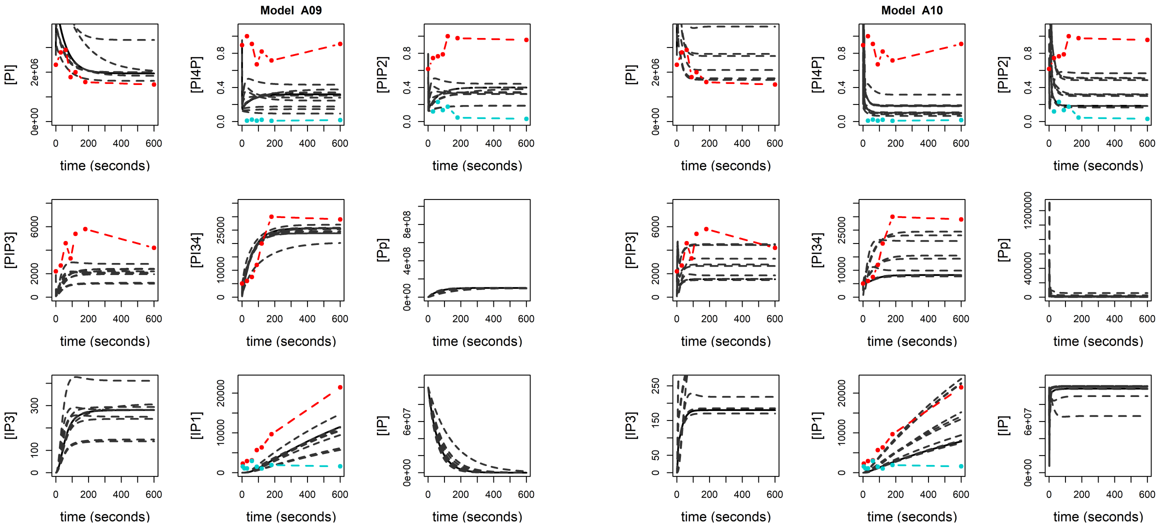

Figure N: Model A09 (left) and A10 (right) predictions (black) for the effect of the inhibitor Gsk-A1, compared to the original experimental data (bright red) and data under the effect of the inhibitor (cyan). Simulations are based on the parameter values from the 10 'best' fits. Solid line denotes simulation with lowest cost function.

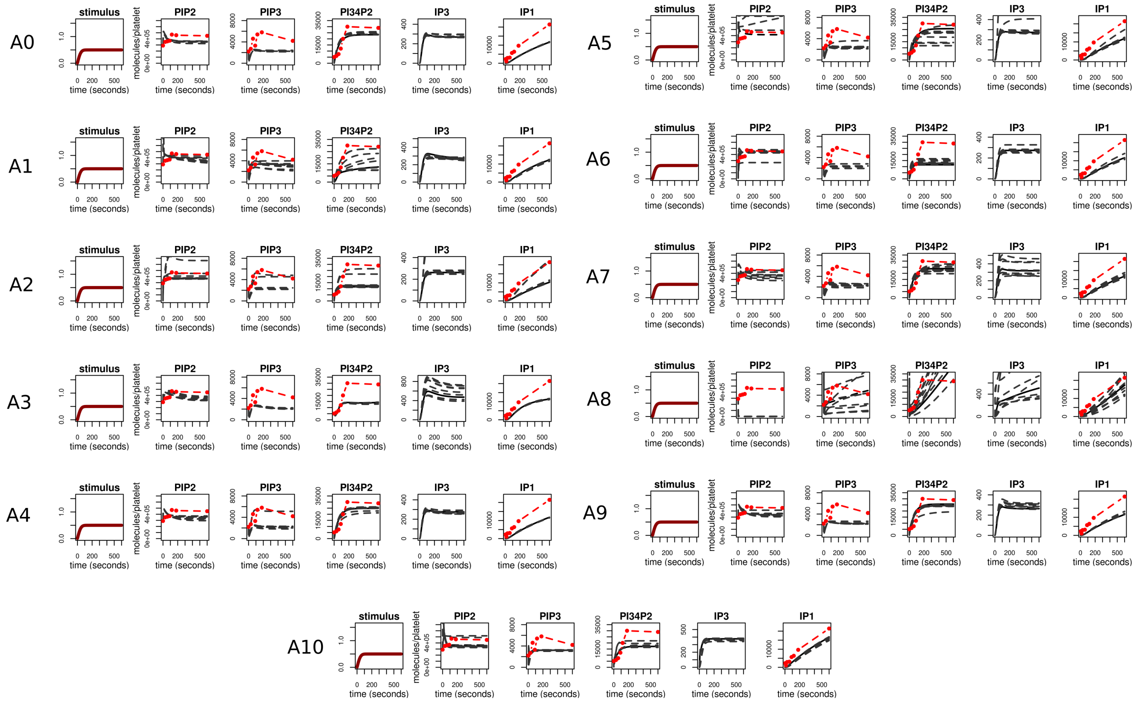

Figure O: Model predictions for a reduced stimulus. Simulations are based on the parameter values from the 10 'best' fits. Solid line denotes simulation with lowest cost function.

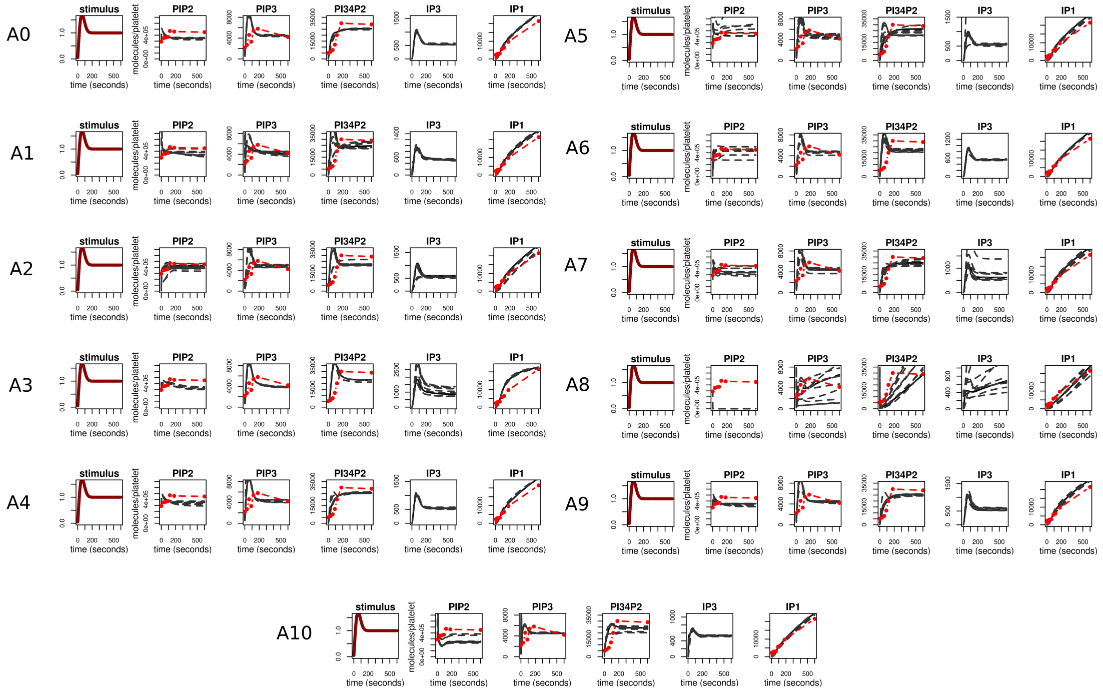

Figure P: Model predictions for a stimulus with an early peak. Simulations are based on the parameter values from the 10 'best' fits. Solid line denotes simulation with lowest cost function.

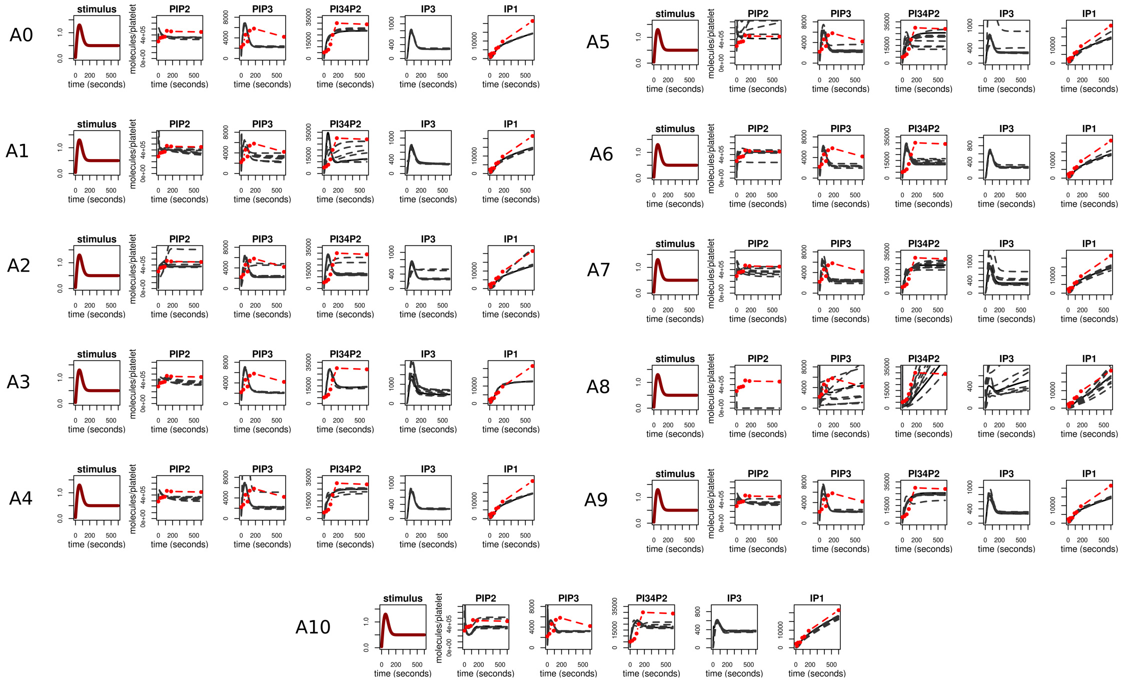

Figure Q: Model predictions for stimulus reduced in magnitude and with an early peak. Simulations are based on the parameter values from the 10 'best' fits. Solid line denotes simulation with lowest cost function.
